# Analectic

**Published:** 1834

**Authors:** 


### MISCELLANEOUS INTELLIGENCE.

### ANALECTIC, ANALYTICAL, AND ORIGINAL.

### I. Analectic.

*Ad Sylvas Nuncius.*

1. *Seat and Nature of Gonorrhœal Orchitis.*—The *Journal Hebdomadaire*, for the 17th of May last, contains an interesting memoir on this subject by Dr. Marc-Moreau. The author maintains, we believe with justice, that hernia humoralis consists of an acute inflammation of the vasa deferentia, of the epididymis and tunica vaginalis, and that this inflammation rarely extends to the substance of the testicle The truth of this view he considers demonstrated—1st. By anatomy, which teaches us that the testicle enveloped by a dense, elastic, and fibrous membrane, could not acquire three, four, five, six, and ten times its normal size, without being disorganized. 2d. By clinical experience, which has shown the existence of fluctuation, especially at the commencement and termination of the disease. 3d. Finally, by post mortem examinations, in which the testicle, which was supposed to be diseased, has been found healthy; whilst the epididymis and tunica vaginalis have constantly presented pathological alterations, and that in the cavity of the serous membrane a fluid of variable colour, consistence and qualities have always been found.

2. *Properties and Effects of the Digitalis Purpurea.*—The Nos. of the *Archives Génerales* for January and February last, contain an interesting memoir by M. Jonet, on the digitalis purpurea. From all the facts which this writer has collected, he infers—

1st. That the powder and watery extract of digitalis, (he prefers the latter,) may be administered in doses as high as sixteen or eighteen grains, commencing with one grain, and daily increasing the dose, without in most cases producing marked disturbance of the digestive functions. That the alcoholic extract cannot be depended upon, and that the etherial extract is the most uncertain preparation. The infusion he considers as the most active preparation, and the dry plant to be preferable to the fresh leaves.

2d. That in a great majority of cases, digitalis employed in powder, aqueous extract, and especially in infusion, exerts an irritating action upon the digestive organs, always evinced by colic, diarrhœa, nausea, and vomiting. That the property of the digitalis in rendering the pulse slower is indisputable, and that the gastro-intestinal irritation does not prevent this result. That the respiration may be affected by the remedy, dyspnœa most frequently disappearing as the pulse diminishes in frequency. That a distinct disorder of the nervous system is rarely observed after the administration of digitalis. That the digitalis really possesses hydrogogue properties; and that the decoction of this plant in the dose of from two to four ounces, applied to the abdomen, is a powerful diuretic, very preferable to the internal administration of other diuretics, because it may be employed in all cases of gastro-intestinal irritation.

3d. That palpitations of the heart, which are most frequently the prelude to a severer affection, ordinarily yield to the use of digitalis. That anasarca and ascites may be relieved by the judicious use of this plant, and that the success attributed to it in mania, hæmoptysis, advanced phthisis, scrofula, and many other diseases is certain.—*Archives Gén. March,* 1834.—*Ame. Journ. of the Med. Sciences.*

3. *Treatment of Ileitis.* By Wm. Stokes, M. D.—Laxatives are to be employed in enteritis, on the same principle that emetics are used in cases where corrosive poison has been taken into the stomach. We are not to expect to be able to cure the disease by the use of laxatives, nor are we to have recourse to them in every case; we employ these remedies where we have decided evidence of the existence of offending matter in the bowels. We may meet with a case in the early stage under such circumstances, that the removal of the irritating matter by judicious purgation may completely relieve the patient, and this, I believe, is the foundation on which the superstructure of the British purgative practice in ileitis and tabes mesenterica was raised. It was concluded, that a laxative treatment, which had on many occasions succeeded in removing the first symptoms of the disease, would necessarily cure it in all stages and cases. This, I need not tell you, is wrong. Whenever you give purgatives or laxatives in enteritis, bear this in mind, that the effect which you have to produce is to be brought about at the least possible risk. If you can unload the bowels with a little castor oil or rhubarb, or some mild neutral salt, it is much better than to have recourse to calomel, or scammony, or colocynth. As a general rule, drastic purgatives must be avoided in inflammation of the mucous membrane of the intestines. The school of Broussais committed an error, on the one hand, by never admitting the use of laxatives, and British practitioners have been wrong, on the other hand, by giving too much purgative medicine. The error of the latter arose from looking always upon purgatives as antiphlogistics, which they are certainly, so far as they contribute to relieve inflammation by causing an increased secretion from the intestinal mucous surface. But this increase of secretion can be produced only by stimulating the organ to which they are applied; and hence, before they can become general antiphlogistics, they must, of necessity, be local stimulants. Further; if in a case of inflammation of the digestive tube you prescribe a purgative, and it fails in causing an increase of secretion, it will add considerably to the existing inflammation. It is, however, of very great importance that there should be no accumulation of offending matter in the bowels; and hence, when you find a degree of fulness in the belly, and the dejections scanty, you should always give a laxative, and follow it up by the administration of a narcotic. By using enemata, you can do a great deal of good, and this without any injury to the digestive tube; and I think they may be always employed with benefit in disease affecting the ileum. Recollect, gentlemen, what I wish to impress upon you respecting this part of the treatment is, that laxatives are to be employed in ileitis as one of the means of cure; but you are not to expect that a cure by the use of these alone will always be a matter of constant occurrence. It is true, that many cases presenting symptoms of enteritis, have, in the beginning, yielded to laxatives; but it is true also, that horrible mischief has been done by their continued or indiscriminate employment.

A few observations now with respect to bleeding. There is in simple inflammation of the mucous membrane of the intestines this peculiarity—it very seldom happens that it is necessary to use the lancet. The whole class of intestinal inflammations is so generally accompanied, even in the early period, with marked prostration and a typhoid condition of the whole system, that general bleeding is very seldom employed. But when the disease is recent, the constitution vigorous, the patient young, the skin intensely hot, and the pain violent, (a combination of circumstances which is not of very common occurrence,) you may employ the lancet with safety and with great advantage to your patient. But what I wish to impress upon you is this—you must not expect to cut short an attack of enteric inflammation by general bleeding. Over inflammation of mucous membranes in general, but particularly of the intestinal mucous surface, the lancet has comparatively but little direct power; it is in the inflammatory affections of parenchymatous tissues and serous membranes, that we generally observe the most brilliant and decided effects of venesection. Neither can you, as in parenchymatous inflammation, bleed a second and a third time with benefit. In cases of inflammation affecting the mucous membrane of the intestinal canal, your are to look upon venesection as a preparatory step to leeching. Where the pain is violent, the fever high, the attack recent, and the constitution strong, you will do well to bleed; but only bleed once, and then apply leeches in abundance over the suffering organ. There is nothing of more importance, nothing of such decided value, as bleeding by leeches in inflammation of the mucous membrane of the intestinal canal, and here we arrive at a fact, the explanation of which is involved in much obscurity. A patient is attacked with inflammation of the mucous membrane, and glands of the digestive tube, twelve or twenty leeches are applied to the integuments of the abdomen, and their application is followed by extraordinary relief. This is a very curious fact when we consider that between the place where we apply the leeches, and the tissue which is affected, there intervene skin, cellular membrane, superficial fascia, cellular membrane again, deep-seated fascia, muscular substance, cellular membrane again, two layers of peritoneum, and muscular substance enveloped in cellular tissue. Yet, notwithstanding this extraordinary succession of tissues, it is an undeniable fact, that the application of a dozen leeches to the surface of the belly will frequently cut short an intestinal inflammation, or materially diminish its intensity. Here is a fact, the explanation of which is extremely difficult; and I tell you candidly, I cannot explain it. The school of Broussais attempt to explain it as follows. They state that it is a constant law of the economy, that there is a strong sympathy between the internal parts and their respective integuments, but they do not say why this sympathy should exist. We frequently, however, observe facts confirmatory of this law; you are aware that it often happens that, in cases of the deep-seated muscular phlegmon mentioned by Mr. Crampton, in abscess of the liver, and in empyema, we have a swelling of the integuments, showing the existence of a sympathy between the integuments and the internal organs.

In treating a case of inflammation of the small intestine, I think you may generally commence with the application of twelve or eighteen leeches over the ileo-cœcal region. The ordinary result of this application is, that the pain and tympanitis are reduced, and the thirst diminished; but the patient still has fever, and you are to bear in mind that the mere subsidence of pain does not imply the removal of the disease. We may modify the character of an ileitis very considerably by a single application of leeches, but we are not on that account to expect that we shall be able to remove the disease entirely. In general it is necessary to apply them two or three times, lessening the number at each succeeding application, and taking care that they are applied in the proper place, that is, midway between the umbilicus and the crest of the ileum. Many practitioners are afraid of employing leeches in the advanced stage of this affection, in consequence of the great debility, which characterizes the advanced stage of this, as well as inflammation of every other part of the digestive tube. But though I am quite of opinion that the school of Broussais is wrong in using them at any period, still I think they may be employed even where the disease is advanced, *particularly if they have not been used before*, and I have frequently seen leeches applied with advantage as late as the twelfth day. I have employed them myself in the Meath Hospital, as late as the ninth and tenth days, with decided benefit. Many physicians on the continent are in the habit of treating inflammation of the digestive system by the application of leeches to the anus, and this is said to have a very good effect, and the number of leeches required is smaller. In disease of the great intestine accompanied by diarrhœa, tenesmus, and tormina, I think this is an excellent mode, but when the disease is in the upper part of the tube, I prefer applying them to the belly over the situation of the inflamed organ.

Now with respect to internal medicines. In this disease every thing that is administered should be given with the view of removing irritation, and for this purpose I know no better preparation than a combination of ipecacuanha and opium, as in Dover's powder. The exhibition of the compound powder of ipecacuanha is attended with decided advantage. You are all aware of the long-established use of ipecacuanha and opium in diseases of the intestinal canal, and I think there can be no doubt that they possess considerable utility. With this I generally combine some mild mercurial; the best you can employ is the hydrarg. cum cretâ. Give two or three grains of each every second or third hour, as the case may be, and you may continue this for several days. Where there is no diarrhœa, and the bowels have a tendency to be constipated, it will be necessary to order, every second or third day, a mild laxative, a little manna, or rhubarb, or some castor oil; you should insist on the daily use of enemata, and if they answer the purpose sufficiently I would advise you to be sparing of the use of laxatives by the mouth. In addition to these remedies, I am in the habit of giving a considerable quantity of gum Arabic, which appears to have an extraordinary efficacy in disease of the small intestine. I look upon it as peculiarly valuable in the diseases of children. The ordinary mode of prescribing it is to give a certain quantity of gum water. If this is sufficient, you should order half an ounce or an ounce of the gum to be dissolved in a pint or quart of water, which the patient is to use during the day. After the use of the hydrarg. c. cretâ and Dover's powder, this has a decided value in the treatment of ileitis.

In this way, by leeching, mild laxatives, prescribing mercury with chalk, and compound powder of ipecacuanha with gum water, your patient begins to improve. The tenderness of the epigastrium disappears, the tongue begins to clean, the fever diminishes, the thirst goes off, and appetite returns. This is the favourable termination. When the patient is of a weak and delicate habit, it is of great importance to pay particular attention to supporting the strength, *even from an early period of the disease.* In such a case, after the first week, the physician who neglects the proper means of supporting his patient's strength does wrong, and it has been justly remarked, that a practitioner will be right in supporting the general strength, at the same time he is employing local antiphlogistics. It is in steering clear between these two opposite dangers that the judicious practitioner is seen; he does not allow his patient to die of inanition, while at the same time he takes care to remove local inflammation. I have seen several experienced physicians prescribe leeches to the abdomen on the same day that they ordered the patient to have chicken broth, and even a little wine. There is nothing improper in this; an experienced practitioner, who has his eye merely on the local inflammation, is apt to fall into the error of overlooking the constitutional debility, and allowing it to steal upon him. He finds very little difference between the appearance of his patient this day and the next, and thinks the slight increase of debility undeserving of an attention. At last his patient begins to sink visibly, he gets alarmed and has recourse to stimulants, but it is now too late. Besides, there are several articles of diet which support strength, without increasing inflammation, as for instance, chicken broth, sago, arrow-root, strained rice, &c. These do no harm, and they prevent the patient from falling into a dangerous typhoid condition. Let us look at this in another point of view. Suppose you are called to a child who is said to have had an attack of worms, or bilious derangement, or that his bowels were costive, and purgatives were given, that the discharges were found to be bad, and more purgatives were administered; or suppose you are called to a child of a weak scrofulous habit; who has been taking large quantities of purgative medicine, for what has been termed *derangement of the bowels*, and you find the little sufferer with pale shrunken face, a black circle round his eyes, cold extremities, rapid faltering pulse; great thirst, and evident symptoms of increased cerebral excitement; the little arms and hands are as cold as death, but the belly burning, tympanitic, and very sensible to pressure; and when you compare the radial artery with the femoral, as it turns over the pubis, you will have some conception of the excited condition of the abdominal vessels; and in addition to this train of morbid phenomena, you find there is suppression of urine.—Are you to attack these symptoms with antiphlogistic means? No; the first thing you are to do, is to prevent any further mischief, by totally inhibiting every kind of purgative medicine. You are next to consider carefully what the best line of treatment to be pursued is, for here you are under circumstances of difficulty, and have a great many prejudices to contend with. What I find generally to be most successful is this. I begin by taking proper steps to support the strength, ordering the patient to take chicken broth, arrow-root, or jelly; the extremities are to be wrapped up in warm flannel; and if the patient is sinking, and has his mouth and teeth crusted with black sordes, a little wine, watching its effects. If it produces sleep, if the pulse comes down under its use, and the fever is not increased, it will do a great deal of good, and you can gradually increase the quantity. Always bear in mind, that there is a certain period in all inflammations, in which stimulants prove to be antiphlogistics, a circumstance which has been overlooked by the school of Broussais. So far with respect to constitutional treatment; but what will you do with local disease?—The application of blisters is of decided use, nay, I have seen a few leeches very effective. Apply a blister to the abdomen, and dress it with mercurial ointment, at the same time you may employ frictions with mercurial ointment: you will also swathe the belly with flannel, so as to keep up a comfortable temperature. In this way you will be able to do a great deal of good. You will also prescribe hydrarg. c. cretâ, with Dover's powder; and if the bowels are confined, emollient injections. By steadily pursuing this plan of treatment, you will often rescue from imminent danger a case which would prove fatal under the purgative plan, and you will add greatly to your own reputation.

There is one form of this disease in which diarrhœa is a prominent symptom, where there is purging from the very commencement. On this form I am anxious that you should have clear ideas. In cases of this kind there is a copious discharge of fluid matter from the bowels.

In the majority of cases, you may lay down this law, that where there is a decided irritation of any secreting organ, increased discharges from the surface of that organ give more or less relief. Suppose two cases of hepatitis; in the one we have no secretion of bile, in the other the secretion is copious; the latter is certainly most favorable. Again, suppose two cases of bronchitis; in one there is a copious expectoration, in the other it is extremely scanty; now every medical man knows that the former is more easily managed. The increased secretion of any organ in the early stage is to be looked upon as a relief to the inflammation. The practical inference to be deduced from this is, that we should be cautious in adopting any means of arresting this discharge, as it is one of the modes which nature employs in relieving the irritation of a suffering organ. Well then, suppose you have a case of enteritis, and that on the first or second day diarrhœa sets in, what does the routine and systematic physician do? He gives chalk mixture and opium with tincture of kino and catechu, and what is the consequence? The belly becomes tympanitic; the pain is increased, and even peritonitis may supervene; this is one result of the increase of inflammation; or the breathing becomes difficult, and the patient gets bronchitis or pneumonia. Diarrhœa occurring in the early period of this disease is not to be interfered with, except when it gets to such a height as to threaten the patient's life; and where it increases his sufferings by the frequency of the discharges. In the first week or fortnight, when there are only three or four discharges, or even five in the twenty-four hours, I believe it is better not to interfere by prescribing direct astringents; *but in the advanced period, when the powers of life are low, or the discharges very copious*, then the physician comes to the assistance of nature with just reason, and in such cases you should always interfere.

The best mode of managing diarrhœa of this kind is to employ small, frequently repeated doses of Dover's powder, with anodyne injections. And here I may mention briefly, to such of you as have not seen them used, the best way of employing them. As these injections are used on a different principle from the common, the latter being intended to empty the great intestine and be discharged, the former to be retained, we are consequently to make the basis of our anodyne injection in such a manner, that it will not prove stimulant from its bulk, or from any irritating substance it may contain. Mucilage of starch, new milk, or linseed decoction may be used as the basis, and the quantity taken for one injection should never exceed three ounces. To this, for an adult, you add from fifteen to thirty drops of tincture of opium, for it is a curious fact connected with this subject, that opium given by the rectum has frequently been observed to exercise a much more powerful effect on the system than when an equal or even smaller quantity has been taken by the mouth. The rule then is, that when you first make trial of the remedy in this manner, feel your way cautiously, and if you find that yonr patient bears ten or fifteen drops, you can increase the quantity on repeating the enema. An eminent practitioner of this city thinks the narcotic effect of opium by the rectum much better marked than by the mouth, and I believe this to be true in many instances. I believe the administration of opium in this way requires a good deal of caution. I recollect the case of a man who had been for a considerable length of time in the habit of using laudanum in large quantities, and was, in fact, a regular opium eater.—During an attack of illness he got an injection containing sixty drops of laudanum; this produced, in a very short time, symptoms of decided narcotism, from which the patient never recovered; in fact, he died, with every appearance of being poisoned by opium.

There is another fact with respect to this disease, which I would have you bear in mind; that, under certain circumstances, inflammation of the small intestine will produce a remarkable tolerance of opium. This applies not only to the advanced stage of enteritis, but also to many other forms of disease. Some time since I made a series of clinical experiments with the view of ascertaining the power which opium possesses in relieving inflammation, and the result has been, that in many cases where the powers of life are so low that we cannot have recourse to the lancet, or any kind of depletory measures, opium alone furnishes us with a powerful means of subduing inflammatory action.

When we come to treat of peritonitis, I shall have occasion to speak of the good effects of very large doses of opium, particularly in that form of the disease which results from intestinal perforation. My first trials of this remedy were in affections of serous membranes, and to this I was led by some interesting clinical experiments made by Dr. Graves. I next tried it in diseases of mucous membranes, where antiphlogistics were inadmissible, and here, as in the former cases, I had many proofs of its great efficacy. I shall state the particulars of a very remarkable case. A young gentleman, a pupil of mine, and a member of the class at Park street, of an irritable habit, was attacked with intense inflammation of the mucous membrane of the intestines. He had a high degree of fever, and his thirst was so insatiable, that for two days he never ceased calling for drink. His pulse was weak but rapid; his tongue red and pointed; respiration very much hurried; but the stethoscopic signs of disease of the lung were absent. His belly was exceedingly tender on pressure; and he had another remarkable symptom—constant smacking of the lips. The case, as you may perceive, was one of severe gastro-enteritis, and it was treated in the ordinary mode, by leeches, cold water, &c., but the disease showed great obstinacy, and at the end of a month the patient was evidently in a state of imminent danger. At this period a curious revulsion took place; the chest became engaged, and the patient got bronchitis. For this he was blistered, and took the decoct. polygalæ with large doses of carbonate of ammonia, under the use of which he recovered. The bronchitis disappeared, but was almost immediately replaced by symptoms of intense gastro-enteric inflammation, thirst, quick pulse, tympanitis, low delirium, and subsultus tendinum. In the course of two or three days diarrhœa came on, becoming more profuse as it advanced. The first day he had four discharges, the next eight, and thus went on increasing until there was a constant discharge of thin fluid matter from the anus. The patient was quite run down, and on three different occasions his friends thought him dead. Having made an unsuccessful trial of various stimulants and astringents, I determined to try what might be expected from large doses of opium. The patient was dying, and it was necessary to do something instantly, which would be likely to arrest the diarrhœa. I ordered a grain of opium to be given every hour; on the first day he took twelve grains with apparent benefit, the next day he took six, the same quantity on the third day, and on the fourth the diarrhœa had so much diminished, and the young gentleman was so much better, that I thought it might be safely omitted. From this period my patient recovered rapidly. I would not bring forward this case in proof of the efficacy of opium if there were not many others of a similar kind; and I have no doubt that this was a cure effected by the use of opium in large doses.

In the treatment of this disease by opium, there is one simple rule, by observing which you will be able to avoid all difficulties, and at the same time have a criterion to judge of the value of the opiate treatment. If the remedy produces the ordinary narcotic effects of such large doses on the system, *it will not do much good.* You begin, therefore, cautiously; and if, after the first or second dose, you find that decided narcotism is produced, or at least more than you would think the quantity given could have brought on, give it up; it will be dangerous. But if he bears one, two, or three grains, or if, after having taken six or eight grains in the twenty-four hours, he appears to be improving, you may then persevere in the administration of opium, and it will be attended with decided advantage.—*Lond. Med. and Sur. Jour.. Feb.* 22*d*, 1834.—*Am. Jour. Med. Sciences.*

4. *Treatment of Diarrhœa.* By Wm. Stokes M.D.—Diarrhœa is the frequent passing of stools of a more or less watery consistence, and which may, and generally does occur without fever. This affection may be considered to arise under three different circumstances, but, in point of fact, every form of the disease may be referred to a single cause, as there is no essential difference in the actual nature of the circumstances by which they are produced. A patient, for instance, takes a quantity of indigestible food, this produces irritation in the gastro-intestinal mucous surface, and diarrhœa is the consequence. Another is exposed to cold, or gets wet feet, the mucous membrane of the bowels becomes more or less inflamed, and this terminates in diarrhœa. Again, a patient, laboring under hectic, has profuse perspirations, these go off and are replaced by frequent fluid discharges from the bowels,—here also, the result is called diarrhœa. All these forms are, however, referable to the same cause,—irritation of the mucous lining of the digestive tube.

A man commits an excess at table, eats something that he cannot digest, and gets diarrhœa. If you happen to be called to such a case at an early period, your course is very plain and easy; there is every chance that the affected organ has received (as yet) no material injury, and it is attempting to relieve itself by increased secretion. The indication here is to get rid of the source of irritation as soon as possible, and this is best done by prescribing a laxative to remove the offending matter, and then following it up with an opiate. The simple rule is to relieve the intestine and prevent the liability to inflamation. A mild laxative, followed by opiates and demulcents, keeping the patient on a low regimen for a few days, & in a warm temperature; this is sufficient for the management of the first term of the diarrhœa. In point of fact, the principal thing, which the practitioner has to do, is to watch the patient, and take care not to permit the inflammatory action to become developed. It is in such cases as these that the expectant medicine is of value. What you are to direct your attention to, is the state of intestinal surface. If a patient gets an attack of pain, if his belly becomes tender on pressure, if he is more or less feverish, you may be sure there has been some mischief done. If, on the contrary, the diarrhœa yields to the exhibition of a mild laxative and light diet; if the pulse soft and the belly not tender you have no reason to fear. But if the purging becomes more distressing, if the pain is severe, the abdominal tenderness evident, the thirst and restlessness continue unabated, it is a sign that the irritation has produced something more than mere increased secretion, and that actual disease of the mucous tissue is setting in. We have now a true inflammatory diarrhœa, which may be looked upon altogether as an enteritis of that kind, in which there is a copious secretion from the surface of the intestine. You observe this leads us at once to the principles of treatment. Here we have fever, pain, frequent morbid stools, thirst, and abdominal tenderness. Well then, what are we to do?—In a case where these symptoms are so severe as to excite alarm, at once begin by applying leeches. Where there is merely evidence of intestinal irritation caused by indigestible food, give a laxative, and follow it up with an opiate; where, in addition to the ordinary symptoms, you have fever, pain and tenderness, never omit the application of leeches. Many a time have I seen cases of this kind, in which chalk mixture and astringents not only failed but even caused additional suffering, speedily and completely relieved by the application of a few leeches. In using leeches, too, we are not, like the practitioners who trust to astringents, playing at the game of double or quits; nor do we stop the purging by exchanging it for something else equally bad, or even worse, for a peritonitis or a bronchitis for instance; *by removing its cause we not only check the diarrhœa; but we obviate any tendency to a metastasis of inflammation to other tissues, and our mode of cure has at once the merit of being successful and safe.*

A patient who has had an attack of the diarrhœa should have his belly swathed with flannel;—this should never be neglected. He will also experience a great deal of benefit from the use of the hip bath and occasional opiates. Give also a combination of rhubarb and Dover's powder, and you will find that it will do him a great deal of good.—This is the remedy which Rhæderer & Wægler found to be of extraordinary advantage, in the mucous fever with diarrhœa which ravaged parts of Germany in the last century. Give two or three grains of each every second or third hour; and increase or diminish each of the ingredients according to circumstances, increasing the Dover's powder where the indication is to remove pain and irritation, and increasing the rhubarb where you wish to produce a laxative effect. This combination forms a remedy of decided value in enteric inflammations: it has been much used in such cases by Dr. Cheyne, and I have repeatedly employed it in the Meath Hospital with marked advantage. You are also to bear in mind that though the principle of treatment in this disease is to remove its cause and put a stop to the purging, still you are in no case authorized to give it a sudden check by astringents in the early period. I gave the reasons for this at my last lecture, and showed that it was based upon a general law of economy. If an organ in a state of inflammation pours an increased quantity of secretion, *it is the mode in which nature attempts to give relief, and if you suddenly arrest this secretion, the probability is that you will excite more inflammation in that organ, or cause a metastasis in other parts.* This is particularly the case if inflammatory fever exists. You must also attend to your patient's diet. Your object here is to support him on such a diet as will require but little digestive power, and will not produce large collections of fæcal matter in the bowels. Jellies, arrow-root, chicken broth, and mild farinaceous food are the only things that can be used with safety, until the intestinal irritation has subsided.

By pursuing this plan of treatment with steadiness and decision, you generally succeed in cutting short the disease. In some cases the diarrhœa will run on to the chronic stage, just like the gleet which follows gonorrhœa; and this is to be looked upon as the apyrexial period, in which antiphlogistic remedies are no longer admissible, and when you may employ stimulants & astringents with effect. The best way to manage this form of the disease, is to make your patient use warm clothing, or even temperature, and mild nutritious diet; to prescribe the vegetable and astringent tonics, the hip-bath, and the occasional use of mild laxatives, followed by an opiate. In this way, after some time, the disease generally goes off, and the patient recovers his strength.—But it may happen that this gleety discharge will continue unabated; it is running the patient down, and he wants some decided remedy to check it. Now the remedies which appear to have the greatest power in stopping this discharge, are the metallic astringents, and the turpentines and balsams, combined with some of the preparations of opium. It is a curious and interesting matter to consider how these remedies act. They are a class of medicines which exercise an extraordinary influence over discharges from the mucous surface, in a way we do not understand, but the effect is to arrest these discharges. In a case of ophthalmia, accompanied by copious secretion from the conjunctiva, or in a case of chronic gonorrhœa, we know there is nothing more beneficial than metallic astringents and balsams; and we are also aware of the great value which turpentine and balsam copaiba possess in checking the increased expectoration of a chronic bronchitis. In diarrhœa, also, they have the same power; they check inordinate secretion, and remove the morbid condition of the mucous membrane on which it depends, by some effect produced on the surface of that membrane, but in what manner this is accomplished we know not. In severe cases of this gleety discharge, one of the most certain remedies we can employ is acetate of lead. You will seldom have occasion to use this or any of the other remedies alluded to, in the case of a healthy person, because the disease will seldom pass into this second or gleety stage, but if it should, and that it is running down the patient, it behoves you to check it as soon as possible, consistent with safety.—Give then the acetate of lead in free and repeated doses, and it is singular to mark what quantities of it patients under such circumstances will bear without any bad consequence ensuing. Hitherto many persons have been afraid to employ it in large quantities, from fear of producing painters' colic; but at present it is known that this disease is to be attributed to the absorption of the carbonate of lead in almost every instance, and that the acetate is comparatively harmless. On this point I can mention one interesting fact, namely, that I have been in the habit of using it constantly, and in considerable doses, for the last six years, and I cannot bring to my recollection one single instance of colic produced by it. One patient in particular, who was under my care, took it in very considerable doses for six weeks, and without any apparent injury. The only cases, in which I have seen the acetate of lead act as a poison, were those in *which it had been used as an external application.* Whether it be that this remedy is more pernicious when employed after the endermic mode, or whether, when applied to the skin, it attracts carbonic acid from the air and is converted into a carbonate, I do not know, but of this I am certain, that where bad effects have followed the employment of the acetate of lead, they have been brought on by its external use. I generally use this remedy in the form of pill, prescribing two grains of the acetate of lead and a quarter of a grain of opium, three times a-day. With the same intention you may employ the turpentines & balsams, which have a powerful effect in checking mucous discharges. Dr. Pemberton, in his work on Abdominal diseases, speaks very highly of the efficacy of balsam copaiba; and I have seen many cases where turpentine has had a great efficacy in arresting chronic diarrhœa. You will see, in the works on materia medica, some other remedies which you can employ with benefit in such cases, but I may mention one which is not generally known,—the alkali of the nux vomica. Strychnine was first used in checking mucous discharges by a German physician, and afterwards by Dr. Graves in this city. The cases, in which it proves most successful, are those in which there is a mere gleety discharge, a copious secretion from the mucous surface without any inflammatory action whatever, or if there be, where it is so low as not to produce the least feverish excitement or pain. Cases of this kind, in which strychnine has been eminently successful, have been published by Dr. Graves. Among others is that of a gentleman, who had sudden calls, so that he often had not time to reach the closestool. He passed a quantity of thin jelly-like substance, and then experienced a transient relief until another attack came on. This case was cured by the use of strychnine, one-twelfth of a grain, three times a-day, made into pills with crumb of bread or aromatic confection.

I may mention here, that, in treating gleety diarrhœa in this way, one thing should be always borne in mind;—it is always dangerous to check any copious secretion suddenly, and the danger consists in the liability to metastasis or new inflammation. Never forget this. What generally happens is, that the patient's belly begins to swell, and you have ascites rapidly formed. Now, I have never seen a case do well in which this kind of ascites came on after the sudden checking of a diarrhœa, the patients all died. Another consequence is the rapid supervention of pulmonic inflammation, and here the disease is almost as bad as in the bowels. You will ask how this unfavorable termination may be avoided. The best mode is, while you are arresting the discharge from the bowels, to promote a determination to the surface.—While you are using opiates, and stimulants, and astringents, employ general warm bathing, or the hip bath, dress the patient in flannel, and use mild diaphoretics every night. You will also do right in blistering the belly occasionally. In this way you will succeed in curing the worst cases of this chronic flux, without exposing your patient to the risk of new inflammation, or translation of disease to other organs.

One of the most common forms of diarrhœa is the purging which occurs in cases of phthisis; a physician will be called to treat this as often as any other, and it is of importance that you should have correct ideas with respect to its pathology and treatment. The ordinary opinion is, that this kind of diarrhœa is one of the results of hectic fever, and many practitioners in treating the purging of consumptive patients, overlook the actual condition of the intestine, and only take into consideration the state of the whole constitution, of the hectic state of which, the diarrhœa is looked upon as one of the symptoms. The consequence of this is, that they do not proceed on the same principles in the treatment of this as of other similar affections of the intestinal canal. Now I would impress upon you, that you should always consider the diarrhœa of phthisis as depending in almost every instance on enteric inflammation. There is no fact in medicine better established than this. Persons think it is the hectic which produces the purgation, but I believe the converse of this proposition is often much nearer the truth, and that the constant diarrhœa often produces and keeps up the hectic. If you examine the digestive tube of a patient who has died with symptoms of phthisical diarrhœa, you will commonly find extensive ulcerations in the colon, cæcum, and ileum. In some cases of consumption, where the purging has been very severe, the amount of disease will often be found to be quite extraordinary; I have often seen the whole of the lower part of the tube one sheet of extensive ulceration. I find I have not brought up any specimens of the effect of phthisical diarrhæa from the museum, but will exhibit them at our next meeting. The preparations before us are those which are illustrative of dysentery, but they will convey to you a good idea of the state of the great intestine in the diarrhœa of consumption, for the effects are nearly the same. Observe now, gentlemen, the importance of this fact, and recollect that in treating every case of consumption with diarrhœa you will have constantly to bear in mind this enteric complication. Recollect, also, that one of the best means of stopping it, when other remedies have failed, is a blister applied over the abdomen. I could bring forward several cases in which every thing had been tried without success, when a blister was applied to the belly, and from the time it rose, the patient ceased to be troubled with diarrhœa, and continued so up to the period of death. I do not mean that you should in these cases proceed to attack the enteritis with the same vigour as you would a similar disease in the healthy subject. Generally speaking, I believe this form of enteritis, to be incurable, but it is of importance that you should be aware of this enteric complication in phthisis, and when you are called in to treat such a case you should carefully avoid prescribing any thing calculated to add to the existing irritation.

Before I quit this subject, I wish to make one remark by the way of caution. It not unfrequently happens that a person, labouring under chronic diarrhœa, comes to consult a medical practitioner, and tells him that he has been suffering from this complaint for months, that he has eight or nine discharges by stool in the day, and that he has been under the care of five or six doctors in succession without any benefit. Well, you are determined to have your trial too, and you commence operations by putting him on full doses of acetate of lead. After a week or a fortnight he comes back and tells you he is not a bit the better. You then try turpentine or balsam copaiba—no use. Nitrate of silver—the same result. The man gets tired of you in turn, and perhaps goes to a surgeon to ask his advice. The surgeon examines the rectum carefully, and finds at a short distance from the anus, an ulcer, which he immediately touches with a strong solution of the nitrate of silver. The ulcer begins to heal, and accordingly as it heals, the irritation of gut ceases, and the diarrhœa goes off. The surgeon is extolled to the skies, and the doctors disgraced forever in the opinion of the patient. Now this is not an uncommon case. I have seen several instances of it, and I must tell you I was once mistaken in this way myself. These ulcers are situated close to the verge of the anus, they occur chiefly in persons of broken down constitution, and those who have taken a great deal of mercury. They produce irritation in the colon, tenesmus, griping, frequent discharges by the stool, and, most commonly during the straining, a little blood is passed. During the course of the last summer, I treated a soldier for this affection, who had been discharged from the East India Company's service, (as was stated in his discharge;) for incurable dysentery. I examined the rectum, and finding some ulcers close to the anus, had them touched with the nitrate of silver. Under this treatment a rapid amendment took place; and in the space of three weeks the man was discharged quite cured. Now, are you to make this examination in every case? I believe you will act rightly in doing so in every case of chronic diarrhœa in the male, but the examination is absolutely necessary in all cases under the following circumstances: first, when the diarrhœa has been of long standing; secondly, when it has resisted a great variety of treatment; thirdly, when it is combined with tenesmus and a desire of sitting on the night-chair after a stool has been passed, showing irritability of the lower part of the great intestine; and lastly, when the patient's health does not appear to be much affected as it naturally should be, where there was long-continued disease of a large portion of the great intestine. A patient will come to consult you, who will inform you that he has had eight or ten alvine evacuations every day for the last six months, and yet he eats heartily and looks quite well. Under these circumstances, the cause of diarrhœa will generally be found to be ulceration of limited extent low down the tube, and capable of being quickly and effectually removed by a strong solution of the nitrate of silver. I shall recapitulate all the circumstances under which an examination is indispensable; where the symptoms have been persistent, have resisted a variety of treatment, are accompanied by tenesmus, and where the injury done to the general health is not in proportion to the duration of the disease. I may mention here, that a medical friend of mine has communicated to me the particulars of another case of this form of diarrhœa in a soldier who was invalided on this account, and who experienced sudden and permanent relief from the application of nitrate of silver to some ulcerated spots which were discovered near the termination of the rectum.—*Ibid*, *March* 1*st*, 1834.

5. *Some Points in the Anatomy, Physiology, and Pathology of the Vertebra, Column.*—The bulletin of the "Anatomical Society," in the *Archives Générales de Médecine* for March contains a paper by M. Chassaignac, under the above title, which is worthy of notice, and of which the following is the substance:—

In this paper M. Chassaignac draws the attention of the "Anatomical Society" to the existence of certain osseous tubercles, situated at the base and behind the transverse processes of the lumber vertebræ, on the same line with the articular processes, but a little below them. These little tubercles present the appearance of a mamellated process, especially in the two last dorsal vertebræ, and serve as points of attachment to the tendinous slips of the longissimus dorsi muscle, which has been erroneously described as attached to the articular processes.

The sixth cervical vertebræ also presents another tubercle, which we find, but in a less marked degree, in the other vertebræ of the neck. This tubercle is placed in front of the transverse process, and is the more prominent as the individual is advanced in age; it varies considerably, both in size and form, in some subjects being nothing but a simple osseous slip; in others forming a perfect hook, with the concavity turned forward. This process corresponds with the primary carotid artery, which is placed in front, and a little to the inner side of it, and furnishes so certain a guide to the vessel, that by placing a finger on the eminence, we plunge a scalpel into the artery with the eyes shut, without any previous incision. In consequence of this connexion, M. Chassaignac has given it the name of carotid tubercle. The process can be easily felt through the integuments, at least in the dead body, being only covered by the skin, subcutaneous muscle, cervical fascia, and exterior edge of the sterno-cleido-mastoid muscle. To find it, we have merely to extend the neck slightly, and press the finger on the inner edge of the above-mentioned muscle, two inches above the clavicle; at the same time we should be careful that the neck is perfectly straight, for the least rotation is enough to alter the relations between the artery and tubercle. In the living body the process is not so easily discovered, in consequence of the greater resistance given by the subcutaneous and sterno-mastoid muscles.

In an anatomical point of view, the tubercle of which we speak serves to distinguish the sixth cervical vertebra from all the others, and to determine in a positive manner the situation of different organs in the cervical region. As applied to surgery, a knowledge of this process may serve to direct us with the greatest certainty to the primitive carotid, and in this respect is analogous to the osseous tubercle on the first rib, close to the edge of the subclavian artery. It also presents a surface well fitted as a point d'appui, to compress the vessel in cases of sudden hæmorrhage. In the case of a penetrating wound, with hæmorrhage, inflicted in the neighborhood of this process, the blood may come either from the primary carotid, from the inferior thyroid, from the vertebral artery, or from the posterior or ascending cervical branches. The relations of these different arteries with the process in question may serve to determine the precise source of the hæmorrhage, a question otherwise extremely difficult to resolve. It is immediately below this process that the vertebral artery penetrates into the canal of the cervical vertebræ; the posterior cervical is below, and a little external to it: the inferior thyroid, crossing behind the carotid artery to gain the thyroid body, is also very close to it; the tubercle will, therefore, serve as a useful point *de parte* for the exploration of the surgeon when about to apply one or more ligatures.—Should any difficulty be experienced in seeking this tubercle through the integument, it is immediately obviated by a division of the skin and superficial layer of tissue.—*Lancet, May 24th* 1834.

6. *Experiments upon the Sounds of the Heart.*—M. Majendie having recently drawn the attention of the Academy of Medicine to the above interesting subject, and propounded certain views of his own, which differ most essentially from those usually received, in ascribing the first sound to the shock or impulsion of the apex of the heart during its diastole against the thoracic parietes, and the second sound to the impulsion of the base of the heart during its systole, Professor Bouillaud, who has for many years distinguished himself by his zeal in the promotion of auscultatory medicine, deemed it proper to have recourse to direct experiments similar to those which Dr. Hope performed on asses.

He laid bare the heart of a strong, full-sized cock, having previously satisfied himself by auscultation that its two sounds might be distinctly heard. He then listened to its action at first while enveloped in the pericardium, and then when divested of it; with the naked ear, and with the stethoscope; and not satisfied with one examination, he made several; and the result of these was, that he could always hear quite distinctly the double sound, or tic-tac of the heart, although there was no point of contact between the organ and any part of the thoracic walls. The friction indeed of the heart against the end of the stethoscope caused a particular sound; but this sound, (simply one of rubbing,) was so very different from the tic-tac of the organ itself, that it is almost quite impossible that they can ever be mistaken for each other. When the heart was cut out, by being separated from its attachments, it continued to beat for a few moments; but these beats of the empty organ were not accompanied with any perceptible sounds.

The preceding experiment was repeated twice upon rabbits with the same results; viz. the sounds of the heart were most distinctly heard, although neither during its diastole nor during its systole could it come in contact with the thoracic walls.

In conclusion, the Professor states that the results of his direct examination of the sounds of the heart have confirmed him in the opinion that the double bruit or tic-tac, which imitates so closely the clicks of a valve, is, in fact, to be attributed to the play of the valves of the heart.—*Med. Chirurg. Rev. and Journal Hebdomadaire, No.* 9, 1834.

7. *On the relations of the cranium to the Organ of Hearing.*—Professor Mojon, of Geneva, in a memoir read before the Royal Academy of Medicine, on the 25th of March last, has suggested some novel and very interesting speculations on this subject. Hitherto we have been led to view the cranium only as a safe recipient of the cerebral mass and of its appendages; but M. M. ingeniously supposes that it serves at the same time as an harmonic case, or drum, to the auditory organs. Treviranus, Esser, and others had already observed that the tympanum is not essentially necessary to the transmission of sounds, and that the sonorous undulations may be conveyed to the nerve of hearing by the medium of the cranial bones; but no one, before our author, had attracted the attention of physiologists to the curious relations which seem to exist between certain states of the cranial bones and the power of discriminating musical sounds.

The post mortem examination of Dr. Bennati, first suggested to M. M. the following speculations, and they arose from his observing that the bones of the cranium were much thinner than usual, translucent at many points, and soldered together along the line of the sutures.

A similar condition of the cranial bones has subsequently been found by him in the body of another celebrated musician.

This coincidence of cranial attenuation, and musical endowments, led M. Mojon to consider whether it was possible that the one might be related to the other as cause and effect: and he has been induced by numerous observations to infer that the cranium is by no means quite passive, in the perception of sounds, that differences in the thickness of its walls may have very considerable influence in determining the degree of acuteness of the faculty, and therefore that it may be regarded as a sort of harmonic case which communicates its vibrations to the organs of hearing.

In confirmation of these views our author alludes to the cases of deaf people, who often can perceive very distinctly the sounds of a piano, or organ, by applying one extremity of an iron rod to their forehead; and the other to the instrument; and who may be made to hear what is said to them if only the voice is directed by a speaking trumpet upon some part of the forehead. It is not unfrequently also that a person whose hearing is indistinct, and who chances to wear a wig, can listen with much greater facility when the head is quite bare, than when it is covered.

The curious observations of M. Perier, on patients who had been trepanned, and who were found to hear quite distinctly any sound directed upon the cicatrix, even when both ears were effectually plugged, (vide our last No.) may also be mentioned as illustrative of M. Mojon's speculations.

Comparative anatomy shows that in a number of animals the transmission of sounds to the organ of hearing is assisted by numerous large sinuses, hollowed out in the bones of the cranium. To us it seems by no means improbable that the musical endowments of the feathered tribes may be in some degree at least modified, or influenced by the very attenuated condition of their cranial bones, and by the existence of the elastic lamellæ, which are found between their supernumerary cavities, as well as the passages or canals which extend into the labyrinth.

The only practical deduction from the preceding views, regards the assistance which may possibly be derived from attention to them, in our diagnosis of deafness, when we wish to discover whether it is owing to a palsied state of the auditory nerves themselves, or merely to some defect or injury of the adjunct members of the auditory apparatus. The every day occurrence of a person squeezing his head with both hands to deaden any very loud noise, may very probably effect the desired purpose, as well by interrupting the cranial vibrations, as by the direct obstruction of the auditory passages.--*Journ. Hebdom. No*. 16. 1834.

8. *Structure and Functions of the Skin.*—The *Gazette Médicale de Paris*, for the 8th of February last, contains an analysis of a highly interesting memoir presented to the Royal Academy of Sciences, by MM. Bresshet and Roussel de Vauzeme, on the structure and functions of the skin. In this memoir the authors endeavor to establish—

1st. That there exists in the skin an apparatus for the secretion of sweat, consisting of a glandular parenchyma which secretes this liquid, and of canals which pour it out on the surface of the body. These excretory canals are disposed in a spiral form, and open very obliquely under the scales of the epidermis.

2d. That the organs of absorption differ in some respect from the lymphatic vessels or veins, with which they nevertheless appear to communicate. These organs consist of transparent canals, of great fragility, branching or forming little arches of communication one with another, but in which we can discover no orifice or terminal mouth, which could serve for the purpose of absorption. It is this which leads us to believe that this function cannot take place by a kind of suction, but rather by imbibition, or by a mechanism, analogous to that of endosmose.

3d. That the medium in which these canals are situated, is a substance produced by a true secretion, which being strongly hygrometric, forms a body, by the medium of which the phenomenon, which we still call absorption, is effected; this absorption is only more promptly and more easily produced on mucous surfaces, because on these surfaces, the mucosity, which is analogous in more than one respect to an epidermic body, is less dense and more miscible with the liquids which are to be absorbed.

4th. That the papillary bodies are truly nervous, and the nervous filaments which enter into the composition of each papilla, do not terminate, (by bundles,) so that each fibril is free and isolated, but the nervous ramuscules appear to have terminal arches.

5th. That the papillæ are enveloped in a particular membrane, and in a layer formed by the epidermic corneus substance.

6th. That these papillæ, sanguineous vessels of a much smaller size than the nervous filaments penetrate.

7th. That the different layers of epidermic corneous substances constitute a separate apparatus, composed of an organ of secretion and of a substance, at first placed in fibres perpendicular to the derma, but which afterwards becomes horizontal. The fibres or small stems result from the superposition of small scales; and the epidermis, properly so called, is but a part of these stems situated at the greatest distance from the derma.

8th. That in this epidermic substance, formed of scaly stems, are found absorbing canals and nervous papillæ.

9th. Finally, independently of the apparatus for the secretion of the corneus epidermic substance, there exists in the skin, towards the external face of the derma a small apparatus for the secretion of colouring matter.—*Gaz. Med. Feb. 8th*, 1834.

9th. *Influence of Gravity, and of a Depending Position on the circulation of the Blood, in Health and in Disease.* To appreciate properly the importance oft hese influences, it is proper that we attend for a few moments to the condition of the circulation in different parts of the body in its most frequent attitudes and postures; viz. the vertical or upright, and the horizontal. As the former is the most frequently repeated and longest continued, it may therefore be reasonably believed to exert a more influential operation on the current of the blood than the other. Let us consider the effect of the upright position of the body, (and this, we need scarcely say, includes the sitting, as well as the standing posture,) and we shall at once perceive that the arterial circulation in the inferior extremities is thereby facilitated, while the venous circulation is proportionally impeded. It is not therefore surprising that as the body advances in years, the operation of gravity which is acting constantly, except during sleep, against the venous current, should on many occasions induce engorgement of the veins of the leg, giving rise to varices, and to obstinate ulcers. The circumstance of these being almost peculiar to the lower limbs, can be explained only on the principle we have stated. The condition of the circulation through the head is the very reverse; the arterial current has to ascend against the gravity of the blood, whereas the venous current downwards is favoured by it. Whenever the upright posture is changed for another, say the horizontal, the circulation is very perceptibly affected; the veins of the face and neck become swollen and livid, the carotids and temporal arteries pulsate with greater force, and head-ache and confusion of thought are often induced. These phenomena are still more rapidly and more strikingly developed if the head is lower than the rest of the body. From this example we perceive that the veins of the head and neck are nearly passive tubes; their contractile power is very small, no doubt from its being seldom called into play; and hence they become easily distended whenever the current of their blood is not favoured by gravity. The contractile power of the veins of the upper and lower extremities is much greater; but in the case of the latter it is often much weakened by their almost continued state of distention to which they are exposed.

Now the circulation through the other parts of the body also is affected, and that too very materially, by the influence of the gravity of the blood, but in different degrees according to their situations and positions. As a general truth we may assert, that whenever the venous circulation is favoured by the gravity of the blood under ordinary circumstances, *there* will congestions be apt to take place, or to be much increased when they have already taken place, by any change of the accustomed position; and the reason of this is, that such veins have but little contractile power to aid in propelling their contents. To return to the subject of the cephalic circulation, is it not a fact of daily observation, that scarcely any one is able to continne long in a strictly horizontal position? the head must be somewhat raised above the level of the body, else unpleasant feelings come on, which not only prevent sleep, but may induce dangerous symptoms. It is not improbable that the less free return of the venous blood from the head when we lie down, may have something to do in the phenomena of sleep.—And is it not, in part at least, this cause which keeps up the desire for sleep beyond the requisite period of repose; so that the longer we remain in bed, the longer still we wish to remain? It is not unfrequent to observe in elderly patients who have been, from whatever cause, long confined to bed, a set of nervous and cerebral symptoms supervene, and these may resist every means of relief which may be devised. The perceptive and intellectual faculties become dull and inactive; a state of torpor and apathy, of greater or less degree in different cases, comes on; the patient is unwilling to be troubled with anything, as the answering of questions, and so forth; and when he does return an answer, perhaps it is confused and rambling. These are alarming symptoms, and if they continue and become aggravated we can have no hope of saving our patient.

On dissection of such cases we usually discover some degree of encephalic congestion, and perhaps a trifling effusion within the ventricles. We deem it not improbable that the true source and origin of most of the mischief are to be sought for in the altered state of the cephalic circulation in consequence of the more frequent and longer continued decubitus or position in the horizontal attitude. As it is with the head, so it is with other parts of the body, when they are kept for a length of time in a depending posture. In the chest the stasis of the blood is always more considerable in those parts of the respiratory organs which are lowest; and it has often been remarked, that pneumonia, especially when it attacks those who have been long bed-ridden, very generally affects the base of the lungs. Perhaps some curious and interesting results might be obtained by endeavouring to ascertain the comparative frequency of pneumonia on the left and on the right side, of engorgements of the liver, and of the spleen, in relation to the ordinary position of the patients during their sleep. It is quite possible that the blood may acquire a tendency to accumulation in particular organs on that side which the person usually assumes while asleep.

In our July number, of last year, there is an interesting memoir of M. Piorry, on what he designated "pneumonia hypostatica," or pneumonia arising from a continued state of congestion of certain parts of the lungs, kept up by long confinement in bed. Almost all the cases occurred in old infirm patients, admitted into the La Salpetriere as objects of charity. The mere confinement to bed appeared often to bring on cough and other pectoral symptoms, and these were found to be quite irremediable, if the patients were kept all day in the horizontal position.

Auscultation readily discovered the seat of the pulmonary lesion; the dullness on percussion, and the absence of the respiratory murmur, with the consecutive râles, heard on each side of the spine, showed that it was the posterior part of the lungs which were chiefly affected; and the post-mortem examination confirmed in every case the accuracy of the diagnosis.—[Ed.]

The injurious effects of a depending position are well illustrated in the case of the female mamma, when not properly supported, especially during lactation; the veins become much enlarged and distended, and not unfrequently severe darting pains are felt through the organ, giving rise to apprehensions of the commencement of serious disease. Then, too, the very common malady of hæmorrhoids is another striking example of the influence of gravity on the circulation of the blood; and the phenomena of many uterine affections also afford testimony to its operation: thus numerous cases of inflammation of the womb are induced by the patients too soon leaving bed, and getting up; the change from the horizontal to the vertical position favours the more easy flow of blood along the uterine arteries, while it retards the returning current in the veins: hence, therefore, we may readily explain the occurrence of inflammation or hæmorrhage under such circumstances. Every obstetrical physician knows that it is of paramount importance to enjoin a reclining posture in all affections of the female internal organs of generation.

Again; it is the agency of mere gravity which induces a varicose state of the spermatic veins in men, constituting the diseases of varicocele and cirsocele, and these diseases are invariably aggravated by all causes which are capable of increasing the force of the gravity of the blood, or of relaxing the coats of the blood-vessels, such as exercise, long standing, heat, &c. The use of a well-made and well-applied suspensory affords by far the most effectual relief. But the phenomena which result from the influence of gravity are still more apparent and striking in the extremities of the body. If the hand has been long hanging by the side, especially when it is warm at the same time, the veins become full and distended, every minute ramification can be traced, and the whole volume of the soft parts is greatly increased, so that even a feeling of unpleasant tension may be induced: by merely raising the hand and arm, and keeping it for some time in that position, all these appearances vanish, and the member resumes its wonted condition. This affords one of the best examples of the influence of mere gravity on sanguineous accumulations; and we can readily believe that the upper extremities would very often exhibit the effects of such accumulations, were it not for the free and frequent movements of them in all directions:—In the case of the lower limbs, the movements are much more limited, and their position is almost always unfavourable, except during sleep, to the return of the venous blood; whether we are walking, standing, or sitting, the blood has to rise from the feet upwards against the force of its gravity. Hence it is that the varicose distentions of the veins of the foot, leg, and thigh are so frequent, and especially whenever there is any superadded cause, which may impede the easy reflux of the circulating fluid—the pressure of the gravid uterus, of an enlarged ovary, &c. is well known to be a common cause of such a malady. When the larger veins of the extremity have been varicose for some time, and especially if the patient neglects the proper means of relief, the capillary veins become gradually distended and engorged—the surrounding cellular substance becomes inflamed, hardened, and ecchymosed, in consequence of blood oozing out occasionally from the over-distended vessels, and being infiltrated into the cellular parenchyma. It is under these circumstances that the skin not unfrequently gives way, and ulcers, most painful and difficult to heal, become formed. Having thus briefly glanced at some of the most illustrative examples of the influence of gravity, as a cause of inconvenience and disease, we shall now direct the attention of our readers, for a few moments, to certain maladies in which the influence of this agent is conspicuously observed.

In severe cephalic neuralgias, the horizontal position is often found to augment the sufferings of the patient; and the only attitude in which he can find any rest, is with his head well elevated. We do not mean to imply that these cases are of an inflammatory nature, yet it is very evident that they are much aggravated by any sanguineous congestion in the parts affected. In phrenitis, otitis, erysipelas of the face, the higher the head is kept raised, the more relief the patient experiences; and when any local inflammation, as of one ear, exists, we uniformly observe that the symptoms are mitigated by lying on the opposite side. Ophthalmia has often been translated from one eye to the other, by the person continuing to lie on the sound side when the inflammation was abating in the other, and this alternation of the seat of the disease may be repeated several times, if the physician's attention be not directed to the real cause. The spreading of erysipelas on the trunk appears to be not unfrequently influenced by the position of the patient; the tendency to spread is generally in a direction to the most depending parts—those on which the patient is resting; and rarely upwards, or to a part more elevated than the spot from which it has started. We have already alluded to the frequency of pneumonic attacks of the lower and back parts of the lungs, in patients who have been long bed-ridden, from whatever cause; and it is unnecessary to do more than merely to point to diseases of the rectum, uterus, and male organs of generation, in proof of the influence of position. In the treatment of ulcers of the leg, we are firmly of the opinion that repose of the limb, in the horizontal posture, is by far the most important of all therapeutic means; poultices, lotions, and ointments will often all fail, unless this necessary adjunct be attended to at the same time; and even when the patient is not strictly confined, do we not invariably employ what may be called compensating remedies, viz. strips of adhesive plaster, or rollers from the toes up the whole length of the limb? and the effect of these is well known to be, the taking off the pressure of the superincumbent column of blood from the veins of the foot and leg.

M. Gerdy, about a twelvemonth ago, instituted a number of experiments at the Hospital St. Louis, on the different methods of treating ulcers; different sets of patients were submitted to the different methods, and each method was employed by itself, in order that the results of each might be justly appreciated. Many of the details have been published in the article "Attitude," in the Nouveau Dictionnaire de Médécine. We shall mention a few of them.

When the limb on which an ulcer existed was kept upon an ascending inclined plane, it was found that the sore became pale, the suppuration was diminished in quantity, and a crust soon began to be formed upon the surface, and under this the healing went on more or less rapidly. If strips of adhesive plaster were used, at the same time that the elevated inclined position was retained, the cure was still more rapid: it was by combining the elevation with the use of adhesive bandages, and the entire repose of the limb, that cicatrization of the ulcer was most speedily effected. Several cases of severe contusion were treated on the same plan, with very decided success—the contused limbs being retained in an elevated inclined position during the whole period of the treatment; the decrease of the pain, tension, and tumefaction was sometimes truly remarkable.

M. Gerdy is of opinion, that many white swellings of the joints may be very materially benefited by an application of the principles which have directed his treatment of ulcers. He recommends that the affected limb be kept perfectly quiet, and on an inclined plane, so that the foot is considerably more elevated than the thigh. He is not yet provided with the reports of any cases to prove the correctness of his ideas; but in one case of elephantiasis of the leg, treated by elevation of the limb, and compression at the same time, the result was most satisfactory—the subsidence of the enlargement was very striking.—*Med. Chir. Rev. and Archiv. Générales, Dec.* 1833.—*Ame. Journ. of the Med. Sciences.*

10. *Of the Chemical Properties of the Secretions in Health and Disease, and of the existence of Electrical Currents determined in Organized Bodies by the Acidity and Alkalinity of the Membranes.* By M. Donné.—1. From the whole surface of the skin is secreted an acid humour. The sweat, however, instead of being, as is generally said, very acid under the arm-pits, and around the genital organs, is, on the contrary, as alkaline in these parts as at the toes.

2. The digestive canal from the mouth to the anus secretes an alkaline mucus, except in the stomach, where the gastric juice is very acid. Thus the saliva and the mucus of the œsophagus, as far as the cardia, are alkaline in a healthy state, and become acid only in consequence of disease. From the pylorus to the end of the intestinal canal, the mucus furnished by the mucous membrane itself is alkaline.

3. Serous and synovial membranes all secrete an alkaline liquor in a normal state, which in certain diseases sometimes becomes acid.

4. The external acid and the internal alkaline membranes of the human body represent the two poles of a pile, the electrical effects of which are appreciable by the galvanometer. Thus, in placing one of the conductors of the instrument in contact with the mucous membrane of the mouth, and the other in contact with the skin, the magnetic needle deviates fifteen, twenty, and even thirty degrees, according to the sensibility of the galvanometer, and its direction indicates that the mucous or alkaline membrane takes negative electricity; and the cutaneous membrane positive electricity.

Independently of these two great surfaces presenting opposite chemical states, there exist other organs, the one class of which may be called acid, and the other alkaline, and which produce the same result; between the stomach, for instance, and the liver of all animals, extremely powerful electrical currents are found.

5. M. Donné has observed electrical phenomena of the same kind in vegetables, of which he gives examples, but electrical currents in vegetables are not produced by the acid or alkaline states of the parts as in animals, because the juice of fruits, at least such as M. Donné examined, is throughout more or less acid. Accordingly, however, to the beautiful experiments of M. Biot, the juices which arrive by the pedicle are modified on some part of the fruit, and it is perhaps to this difference of the chemical composition of the juices of the two extremities that the electrical phenomena are to be attributed.

6. The acid humours of the economy may become alkaline, and *vice versa.*

7. Acidity is usually the result of inflammation, properly speaking, which may be produced by sympathy in an organ situated at a distance from the inflamed point. Thus the saliva becomes very acid in inflammation of the stomach.

8. The acid which is developed in inflammation appears to be most frequently the hydrochloric. The presence of this acid produces coagulation of the albuminous part of the lymph, or of the serosity which abounds in inflamed parts. The false membranes in the serous cavities, the albuginous spots of the eye, the coagulable lymph of wounds, the thickenings of certain organs, and many other morbid productions resulting from inflammation, in which there is found by analysis only albumen, more or less coagulated, are owing to this.

*Pus* itself is produced by the action of the acid on albuminous lymph. It is a kind of union of the acid with the albumen. If free acid be not found in the liquids effused on the surface of inflamed organs, it is owing to the humours of the body being very alkaline, and containing sufficient potass and soda to neutralize the acid. In the memoir, however, of which this paper is a summary, M. Donné has cited many cases in which *pus* and even the serum effused into the abdomen in consequence of *peritonitis* were found acid. An analogous case was reported to M. Donné by M. Dumas, and another is mentioned by Berzelius in his treatise on chemistry.

9. The changes in the chemical nature of the secretions react on the different systems of the economy, forming an interesting order of lesions and symptoms in connexion with the etiology, the diagnosis, and even the treatment of diseases. These changes according to M. Donné, produce modifications of the electrical currents which exist between the different organs of the economy.—*Ed. Med. and Sur. Jour. and Jour. Hebdom. Feb.* 1834.—*Ame. Jour. of the Med. Sciences.*

11. *On Cancer of the Stomach.* By William Stokes, M. D.—[Extracted from his Lectures on the Theory and Practice of Medicine, delivered at the Medical School, Park street.] Pathologists are divided as to what is the cause of cancer of the stomach, but the best informed are of opinion that, in those cases of gastric disorganization, which are called cancer or scirrhus, all that can be demonstrated by the knife is referable to the results of chronic inflammation. This is a different proposition from saying, that chronic inflammation *alone* will produce cancer. As yet we know little of cancer; dissection of cancerous organs gives but scanty information; but this seems certain, that, in particular conditions of the economy, an inflammation of the stomach will end in cancerous disease. Here is an excellent preparation of the stomach of a person who died of cancer of that organ. For several years before his death he had a jaundiced look, an emaciated appearance, frequent vomiting, and severe pain towards the termination of the digestive process, a circumstance which denotes disease of the pylorus. He had also hæmatamesis. You see the inner surface in the vicinity of the pylorus presents ulcerations of the mucous membrane and thickening of the sub-mucous of the cellular tissue. The pylorus itself does not appear to be at all contracted, but the parts around it are in a state of extraordinary disease. Look at the preparation again, and say what could bitters, or acids, or alkalies, or tonics have effected in a case of such extensive disease. Here is a stomach, in a state of long-continued chronic inflammation, and exhibiting lesions, which some would designate as cancer of that organ. Now, though I do not know the treatment which this patient underwent, I would venture to say, that he took plenty of the usual anti-dyspeptic medicines. Yet in a vast number of cases, when enormous quantities of these remedies are taken daily, the stomach is in as bad a state as that preparation exhibits, and I feel the more strongly convinced of this, because I am aware that many persons die after having gone through the whole routine of anti-dyspeptic practice, and, when they are opened after death, incurable disease of the stomach is discovered. Here is an example of vast cancerous disease of the stomach; here is a very interesting specimen of chronic gastritis, chiefly representing a most remarkable and circumscribed ulcer at the termination of the stomach. Here you see is the ulcer, with raised, thickened, and introverted edges. Now, in all probability this ulceration was exceedingly chronic, for you perceive nature has been at work with it, and has made some attempts at preparation. It is in such a case as this that patients generally refer their pain to a particular part of the stomach: digestion goes on without any pain until the food reaches a certain point, when acute pain is felt, and this continues until it is relieved by vomiting. The occurrence of this symptom, after an attack of acute gastritis, would lead you to suspect the formation of one or more ulcers, and the persistence of this localized pain should induce you to persevere in employing every means in your power calculated to remove the disease. The preparation which I now exhibit is interesting, as it shows the effect of corrosive poison on the stomach. The patient, to whom this stomach belonged, died in consequence of swallowing a quantity of sulphuric acid; here you see the consequences, the mucous membrane is black and disorganized, exhibiting this ragged appearance. In some cases of malignant fever we have found the stomach presenting somewhat similar appearances; and the same state of the stomach is described by some writers as occurring in cases of intertropical fever. Here is a preparation, which you should inspect, chronic gastritis with a large ulcerated patch in the centre of the stomach. Here is another example of extensive cancerous disease.

A very few words will suffice for the state of the science on the subject of cancer of the stomach. It is very hard, nay, even almost impossible, to draw a line of distinction between the symptoms of cancer of the stomach and chronic gastritis, and I believe it is admitted on all hands that the same causes give rise to both. Long continued irritation will, in one case, produce cancer of the stomach, in another, chronic gastritis. Again, it is admitted by many, that what is called cancerous ulceration of the stomach has no appreciable difference from ulceration in various other organs; and hence some persons have gone so far as to say that there is no such thing as cancer of the stomach, (separately considered,) and that all the cases adduced of it are nothing more than so many forms of chronic gastritis. In the present state of medicine, we are not, indeed, possessed of any data which would enable us to come to a final determination on this question. It is certainly impossible to determine this point; but if there be any thing peculiar in cancerous matter, similar to tubercular or melanotic matter, there is no reason why, under the influence of inflammation, it should not be developed in the stomach, as well as in any other part of the body. But whatever views we entertain on this subject, we must confess that, in the majority of cases, there is a chronic gastritis, and that the principles of treatment which would alleviate the patient's sufferings and prolong life, *are those which are calculated to prevent the occurrence of gastric inflammation.* The more you approximate the treatment of cancer to that of chronic gastritis, the greater comfort will you afford your patient, and the more will you prolong his existence.

The most celebrated case on record of this affection is that of the Emperor Napoleon. He died with extensive ulceration of the stomach, which, of course, was called "*cancerous*," and there was also distinct traces of disease of the liver, the mucous coat of the intestines, and the lungs. His disease was believed by himself to have originated in the stomach, and to this opinion he adhered, notwithstanding the results of some solemn consultations, at one of which his affection was declared to be an "*obstruction of the liver*," with a "*scorbutic discrasy.*" At another it was pronounced to be a "*chronic hepatitis*," and a course of mercury recommended! When we reflect on this, and read in the account by Gaubert, (which you will see in the *Examen des Doctrines Médicales*,) the regimen which was used, and the list of stimulating medicaments employed, you will not wonder at the words of this great man, when he was pressed to take more drugs, to swallow the universal nostrum, mercury, to which he had the greatest aversion. "Your disgusting preparations are good for nothing. Medicine is a collection of blind prescriptions, which destroy the poor, sometimes succeed with the rich, but whose whole results are more injurious than useful to humanity." But he got mercury, notwithstanding, mercury for his "digestive organs;" to "excite the liver;" to "remove its obstruction," and mercury to create bile, and purgatives to remove it; and tonics, and antacids, and stimulants; and he died in torture, and his body was opened, and the stomach was found "*cancerous*."

I should not omit mentioning to you, that in those cases of chronic gastritis, which run on to an incurable stage, the best treatment consists in a careful regulation of diet, in keeping the bowels open by enemata, or the very mildest laxatives, and in avoiding every thing capable of producing excitement. You will also derive advantage from the employment of gentle counter-irritation, and from the internal use of narcotics, which in such cases appear to have a more beneficial effect than any other class of remedies. With the exception of these, I do not know any other kind of medicine you can safely employ; and I believe that, in the majority of cases, you will find that the patients have taken already a great deal too much medicine. Anxious for relief, and urged on by the hope of obtaining some remedy capable of alleviating their sufferings, they have recourse to every grade of quacks, are persuaded to swallow every kind of drug, and are subject to every form of harassing and mischievous treatment. The diet which you prescribe for such patients should be sparing but nutritive; give the stomach as little to do as will be consistent with the support of life and strength; and you may take it as a general rule in the treatment of all chronic affections of the digestive tube, whether cancer of the stomach, scirrhus of the pylorus, or structure of the intestines, that there are two great principles of general application, preserving a gently open state of the bowels, and allaying inflammatory excitement.—*Lond. Med. and Sur. Jour. Feb. 8th*, 1834.—*Ame. Jour. of the Med. Sciences.*

12. *On Duodenitis.* By WILLIAM STOKES, M. D.—I shall not dwell on the subject of duodenitis, as I shall revert to its consideration when speaking of jaundice, because inflammation of the duodenum is a common cause of jaundice, perhaps the most common, if we take the whole of its causes together. You are not to suppose that I wish to inculcate the doctrine that jaundice is a necessary complication in duodenitis, but it has been proved, that there is an extraordinary frequent coincidence between both, and that jaundice very often seems independent of any mechanical cause, such as an obstruction of the biliary ducts. So far from this that in some cases, particularly those which are produced by, or accompany, a duodenitis, we have intense universal jaundice at the same time that the bile is flowing freely into the digestive tube.

The researches of the immortal Bichat gave the first hint which directed the attention of practitioners to the circumstance, that, in many cases where jaundice had existed during life, there was no obstruction or disease in the liver or biliary ducts, but that in such cases there was always more or less inflammation in that part of the digestive tube, into which the bile was immediately discharged, and this led ultimately to the discovery of the connexion which exists between inflammation of the duodenum and jaundice. In treating of the sympathies which depend upon continuity of surface, Bichat refers to the connexion which exists between the surfaces of mucous membranes and the ducts which open on them, and endeavours to show, that the natural mode of excitement in all secreting glands is a stimulus applied to the surface on which their ducts open. As examples of this, he instances the effect which food and other substances, applied to the mucous membrane of the mouth, have in stimulating the salivary glands; the effect which stimulants, applied to the conjunctiva or nose, have on the lachrymal gland, and many others. Hence Broussais concludes that, when the mucous surface of the duodenum is thrown into a state of excitement, we may have a consequent affection of the liver, for the duodenum bears the same relation to the liver as the mouth does to the parotid glands. That this is frequently the case, I think, is very probable. It is now established, that the cause of the yellowness in what has been called yellow fever, is disease of the upper part of the digestive tube, in which the duodenum is always involved; and that the fever itself, (the typhus icterodes of the nosologists,) has been found to be greatly connected with inflammation of the stomach and duodenum. During the epidemic of 1827, we had in the Meath Hospital a great many cases, which bore a striking resemblance to the yellow fever of warm countries, and particularly in this, that they were accompanied by intense jaundice, and inflammation of the upper part of the digestive tube. You will see in the works of Rush and Lawrence, two of the best American writers on yellow fever, that, of the numerous bodies they examined, there were scarcely any in which the jaundice was found in connexion with liver disease, but that in all cases there was intense inflammation of the digestive surface.—*Ibid.*—*Ibid.*

13. *On Inflammation of the Jejunum.* By William Stokes, M. D —We know very little of the symptoms which characterize inflammation of the jejunum; and it is a curious pathological fact, that this portion of the intestinal tube is of all others the least liable to inflammation. In point of fact, we have no means of ascertaining what are the prominent symptoms of inflammation of the jejunum, because, in almost every case in which jejunitis has been discovered, there has been also extensive disease of the rest of the small intestine. We have cases of simple gastritis; there have been also cases of distinct disease of the duodenum. We may have disease in the lower third of the ilium, unaccompanied by an affection of any other part of the tube. The same thing may occur in the case of the cœcum, colon, or rectum, but it seldom or never occurs so far as the jejunum is concerned.—*Ibid.*—*Ibid.*

14. *On Inflammation of the Ileum.* By William Stokes, M. D.—Inflammation of the ileum is a most important affection, for two reasons; first, in consequence of its extraordinary frequency, and, in the next place, of its insidious latency, the disease generally requiring a considerable degree of tact and experience on the part of the practitioner to make out its diagnosis with certainty. In fever, it is the most frequent of all the forms of intestinal inflammation; and hence Broussais, finding inflammation of the ileum of such constant occurrence in fever, concluded that fever was only symptomatic of intestinal inflammation. Further researches have shown that he was mistaken, and that the inflammation of the digestive tube is, in many cases, secondary; but it is still a circumstance of almost constant occurrence, and in many cases of fever is the cause of death. Now, the portions of the intestinal tube most commonly affected in fever are, the stomach and lower part of the ileum, and the frequent occurrence of this in fever is very remarkable. There are few cases of typhus without it. In some cases of typhus you will, on examination after death, be astonished to find extensive disease of the intestinal canal, which, during life, had not attracted any particular notice, and this you will most commonly find in the lower part of the ileum. So common is it, that Louis says that ileitis is the grand anatomical feature of typhus fever; that is, had he been obliged to pitch on the lesion of some particular organ as giving a character to typhus, he would say that it was ileitis. There are other diseases, too, in which inflammation of the ileum forms the principal complication. In the diseases of children, which go by the names of worm fever, remittent fever, and bilious fever, I believe that ileitis is generally the first affection, and that the fevers are only symptomatic of it. It constantly occurs at some period or other of tabes mesenterica; and I believe, that in many cases it precedes the affection of the mesenteric glands. It is exceedingly common in phthisis. In every case of phthisis, where diarrhœa has lasted for some time, the probability is, there is ulceration in the cœcum, colon, and lower part of the ileum.

Now, what is the nature of this ileitis? This preparation, (*handing one for inspection,*) which I beg of you to hand round, will furnish a very good illustration of the disease. Here is a portion of the intestine exhibiting various distinct ulcerations of different sizes, occupying the situation of the mucous glands. I do not mean to say, that the character of the disease consists in this distinct ulceration; it is an essential disease of the mucous membrane, and of its glands, which exist in great numbers on the surface of the lower third of the ileum, and are called *solitary* and *aggregate*. These glands frequently take on the inflammatory condition, become softened, run into ulceration, and produce extraordinary sympathetic irritation of the whole system. There has been lately a great deal of discussion with respect to the question—Whether disease begins in the glands or in the mucous membrane, and whether we can separate disease of the glands from disease of the mucous membrane. This has been carried to a great extent, and a change has been attempted to be made in the name of the disease, it being entitled *dothin-enteritis* by those who say that the inflammation commences in the glands. But this I think is a mere refinement, and is carrying the thing too far. It is next to impossible for the glands to be affected without involving the mucous membrane, or for the mucous membrane to be affected without an extension of the disease to the glands. We sometimes, however, see the mucous membrane diseased without the glands being apparently engaged; but I think the glands are never engaged without the co-existence of disease in the mucous membrane. In this preparation you see the mucous membrane is just giving way; and here is an actual slough, where the mucous and submucous tunics have yielded to the inflammation. In the lower portion of the ileum we meet with an infinite variety in the size and number of the ulcerations: in some they are very close and numerous, in others there are only two or three detached ones; in some, the whole circle of the intestine is destroyed, and the ulcer is nearly as broad as the palm of the hand. It is interesting to consider, with respect to the pathology of the respiratory and digestive systems, how it comes, that ulceration of the mucous membrane is so much more common in the digestive apparatus than in the respiratory. For one ulceration of the bronchial mucous membrane from acute disease, you will have one hundred of the gastro-intestinal. For this peculiarity we cannot clearly account; but there seems to be more development in the digestive than in the respiratory system, and this over-development produces a tendency to disease. This, perhaps, is an approximation to an explanation of the facts; and to this may be added, that the mucous membrane of the intestines is exposed to the influence of a much greater variety of agents. It is difficult to give an accurate idea of the symptoms of ileitis, as we can only arrive at a knowledge of it by negative evidence, or, as the French term it, "*par voie d'exclusion.*"

In a case of gastritis and of inflammation in the upper part of the digestive tube, the most prominent symptoms are thirst and vomiting. In this affection, too, there is thirst, but it is by no means so urgent as in the former cases, and there is generally no vomiting. In a case of acute gastritis there is always a desire for cold drinks. In this disease there is also a desire for fluids, but the patient prefers them warm. Here you perceive two symptoms, connected with the predominance of disease in the upper part of the digestive tube, are absent—vomiting and the desire for cold drinks.

Now, you are aware, that, in case of inflammation of the colon and rectum, the most prominent symptoms are diarrhœa, tenesmus, and the passing of a quantity of morbid secretions. These symptoms, in a case of ileitis, are either wanting, or they are so slight as to excite but very little notice. If then, in a case of intestinal disease, we abstract the characteristic symptoms of disease in the upper and lower part of the digestive tube from the phenomena of the existing disease; if we find that it presents symptoms which do not properly belong to either the stomach, duodenum, colon, or rectum; we conclude that it must depend on a lesion of the remaining part of the canal, and we are, in this way, led to the diagnosis of ileitis. Let us enumerate the symptoms of an ileitis. In the first place, thirst, without a preference for cold drinks; in the next, absence of vomiting; again, in the early period of the disease, there is generally a tympanitic state of the belly, and the patient seldom complains of pain even in fatal cases. This is a point of extreme importance. There is, however, most commonly a degree of tenderness over the ileum, which you will be able to detect by an accurate examination, and this tenderness presents a remarkable difference from the tenderness of gastritis, both in degree and situation. It is very seldom so exquisite as in a case of gastritis, the patient can bear a considerable degree of pressure, and the tenderness, in place of being towards the epigastrium, is situated between the umbilicus and the crest of the ileum on the right side; here pressure excites pain. The tongue in this affection is generally of a dirty-white, pointed, and red along the edges and tip; the pulse is quick and small, and the face is contracted. As to the nature of the discharges from the bowels they are exceedingly various; there has been as yet no diagnosis founded on their appearance, and in some fatal cases they have been observed to retain an almost perfectly healthy appearance throughout. What would the gentlemen, who draw their diagnosis from chamber pots, say in such cases? I have seen perfectly natural stools in cases, which immediately after have terminated fatally, and where, on examination after death, there was a vast extent of ulceration in the ileum. In addition to the symptoms just recited, the patient most commonly has *fever*, and this presents itself under various forms, frequently assuming the type of a simple continued fever; hence, in a great many cases, *the patient is supposed to labour under merely simple continued fever, and the existence of extensive inflammation of the ileum is entirely overlooked.* In other instances, there is more or less prostration which increases with the progress of the disease, and the fever frequently receives the appellation of typhoid. Under these circumstances, the patient often gets bark and wine, every means is taken to support his strength and remove the typhoid condition of the system, the inflammation of the intestine is exasperated by neglect and maltreatment, the patient dies, and, on dissection, the ileum presents an enormous sheet of ulcerations.

In cases of this kind, where the diagnosis depends as much on negative as on positive circumstances, it is of importance to have a direct sign, by which we may be able to ascertain with some degree of certainty the existence of a suspected enteric inflammation, and I think I have discovered one, which I believe has not been as yet noticed; this is increased pulsation of the abdominal vessels. In many cases of acute inflammation of the brain, the increased pulsation of the carotids has been frequently remarked, and every one sees that, under such circumstances, there is an undue excitement of these vessels, or, in other words, that there is a want of proportion between the action of the carotids and that of the arteries of the extremities. If your finger be attacked by paronychia the same phenomenon is observed, the artery leading to the inflamed finger beats much stronger than the artery of the corresponding one on the opposite side. From these circumstances I was led to conclude that, in cases of acute inflammation of the digestive tube, there would be increased pulsation of the abdominal aorta; and, on following up the investigation, by examining several persons who had distinct and well-marked intestinal inflammation, I found that my conclusions were well-grounded. In such cases, I found not only a remarkable throbbing of the abdominal aorta, but I also discovered that this throbbing was prolonged to the femoral arteries, and that, on the other hand, there was little or no corresponding excitement in the arteries of the upper extremities.

In inflammation of the ileum the patient generally lies on his back, and avoids motion as much as he possibly can, his skin is dry and harsh; he is feverish; he has thirst, but little desire for cold drinks; he scarcely ever vomits; his alvine dejections are sometimes thin and purgative, sometimes figured and natural. But there is one circumstance which is of considerable importance in pointing out the amount of disease, even in cases where patients have considerable diarrhœa, and this is, that the diarrhœa is not sufficient to account for the extraordinary prostration. There must be some cause for the great reduction of vital power besides the mere diarrhœa, and I must state to you that there are few diseases which bring on such rapid prostration as inflammation of this portion of the digestive tube. In the advanced stage of this disease, the patients have cold skin, subsultus tendinum, petechiæ, involuntary discharge of urine and fæces, low delirium, coma, gangrenous ulcerations of the back, sinking of the powers of life, effusions into the head and chest, in fact all the symptoms which characterize the last stage of typhus. Generally speaking, the disease is more or less prolonged, and the patients die of exhaustion, but in some cases the approach of death is more sudden and formidable. Some of the ulcers pass deeply into the substance of the intestine, perforate all its coats in succession, the contents of the intestine escape into the peritoneum, and the patient is carried off by a rapid peritonitis.—*Ibid.*—*Ibid.*

15. *Inflammation of the Ileum in Children.* By William Stokes, M. D.—Inflammation of the ileum is very frequently met with in children, and it is most important that you should be aware of the extreme frequency, as well as the symptoms of this disease, in those little creatures. There is one fact in pathology which seems not to be generally acted on, that there is a class of diseases which are intra-uterine, and with which a child may be born. There are a great many cases of this kind on record, but still I must confess, there is a great scope for investigation, and that our knowledge on this subject is imperfect. I believe that any one who has the opportunity of dissecting a great number of still-born children, or of those who die immediately after birth, would, by examining the state of the different cavities, and publisbing the results of his examinations, earn for himself very great reputation. It is a well-known fact that children may be born with hydrocephalus, with tubercles in the lungs, with acute inflammation of the stomach; nay more, children have been known to be born with chronic gastritis, and with old ulcerations in the ileum and colon. When children happen to be born with gastro-enteric disease, they are puny and weak: the fact of this occurrence is generally overlooked, the case is considered to be one of general debility, and hence most of those children are lost in consequence of their medical attendants being ignorant of the real nature of the disease. It is a very curious fact, too, that when enteric disease occurs in very young children, it is frequently met with without any accompanying fever, and this is a point of great importance. Here is a fact not generally known. A new-born infant has vomiting, swelled belly, contracted features, but at the same time he has cold skin and feeble pulse; he has no distinct symptoms of fever, and a puny and feeble state of constitution appears to be the prominent symptom. He dies, and, on opening the body, you find distant traces of enteric inflammation. The younger the child is, the less will be the chance of fever occurring as a sign of enteric inflammation. It seldom happens that this takes place after dentition, but before it is very common.

Now, what are the circumstances which would enable us to recognise this disease in children who have passed the period of first dentition? If you find the child vomiting, thirsty, with swelled belly, hot skin, a tendency to diarrhœa, and erythematous redness about the anus, you may be sure that there is disease of the digestive system; if the child is restless, and you perceive that the symptoms of irritation of the head are coming on, you will be more certain, and in such cases pathology will inform you that the disease is chiefly in the ileum. In the advanced stage the diarrhœa is lessened, but the belly continues tympanitic, the child exhibits traces of long suffering, and the circumstance of the teeth not being developed gives it the appearance of premature old age, which cannot be mistaken by an experienced eye, and is a sign of long continued and extensive intestinal disease. In some cases, the child gets a common attack of diarrhœa; this is neglected, but after going on for two or three days, symptoms of fever begin to appear. Here we arrive at a practical rule. Where a child has diarrhœa, and after laboring under this for a few days, gets an attack of fever, you may be almost sure that it is a case of enteritis, and that you will be acting wisely in treating it as such. In the opinion of many well-informed practitioners, that form of fever which has been called infantile remittent, is only an example of this disease. In proof of this fact, Dr. Marsh, my friend and predecessor in this school, in his paper on jaundice, makes some excellent remarks on this subject. "There is yet one form of diséase of very frequent occurrence, the seat of which is in the stomach and small intestines. That to which I allude is the *infantile remittent fever*, or, as it is vulgarly termed, the *worm fever* of children. Its characteristic symptoms, if closely analysed, will be found all of them to point to the mucous surface as the original seat of morbid action."—*Dublin Hospital Reports, Vol.* 3.

It would be well for medicine, if the valuable information conveyed in Dr. Marsh's paper was more universally diffused. I feel convinced that many children fall victims to mal-practice under circumstances of this kind. A child gets symptoms of diarrhœa, has irregular or bad appetite, and swelled belly, the disease is called worm fever; he gets a dose of calomel and jalap, and perhaps passes some worms; for when we come to speak of worms, we shall find that disease of the mucous surface is intimately connected with worms, and, in the opinion of one practitioner, worms may be the result of enteric inflammation. Well, some worms are passed; the purgative is again used; the child may not pass any more, or he may pass one or two in the week to encourage the practice. But all the symptoms of intestine inflammation, the diarrhœa, the tympanitis, the thirst, the fever, are supposed to depend upon the presence of more worms, and these are to be evacuated by purgative medicine, and thus the affair goes on until the child falls into tabes mesenterica, or gets sympathetic inflammation of the brain, and dies of hydrocephalus. I regret to add, that in many cases of this find the head alone is opened; a little fluid is discovered in the ventricles of the brain, the doctor's diagnosis of the head is found to be correct, and all parties are satisfied. In cases of this kind, the early application of leeches to the belly, the regulation of diet, keeping the bowels gently open by enemata and mild counter-irritation, would have saved the patient. This is not mere theory, it is but a statement of facts, supported by the experience of practical men.—*Ibid. Feb.* 15*th*, 1834.—*Ibid.*

16. *On Tabes Mesenterica.* By William Stokes, M. D.—The term tabes mesenterica is employed to designate that species of consumption which depends upon disease of the mesenteric glands. The common idea formerly entertained with respect to this affection, and, I believe still to a great extent, is, that the disease first commenced in the mucous glands, and from these extended to the lymphatic ganglia of the mesentery, which in their turn became enlarged, thickened, and less pervious, so that a sufficient share of nutriment cannot be absorbed, the consequence of which is that the patient dies of atrophy and exhaustion. With such views of the case, the principles of treatment consisted in employing a class of medicines called deobstruent, the operation of which was supposed to be efficacious in removing this obstruction, this deposition in the substance of the mesenteric glands, and the enlargement by which it was accompanied. This was, and this, I am sorry to say, is the idea still entertained by many. What is the actual state of the science with respect to this disease? It is found that the glands are certainly changed in their structure, and that they are manifestly enlarged; but this is only a link in the chain of phenomena, for it has been proved that in *the majority of cases the disease is ushered in by enteritis, and that the swelling of the glands is the result of disease, propagated along the course of the lymphatics from the mucous surface of the intestines to the mesenteric ganglia.* This preparation, which I shall send round, will give you an idea of the actual state of the disease. Here is one of the glands which has been cut through; it exhibits the cheesy texture commonly observed in this disease, but you can perceive there are a number of lines running towards each of the glands; these are the engorged lymphatics, which you see correspond with ulcers on the mucous surface of the small intestine. That this is the true pathology of the disease will appear from the following circumstances: First, it has been proved that the glands of the mesentery commonly become inflamed, enlarged, and even suppurate, in cases of inflammation of the mucous membrane of the intestinal canal in the adult. A patient gets enteric inflammation and dies; on dissection we find distinct marks of disease in the intestines, and, in addition to this, we find the glands evidently diseased. Here is one fact. In the next place, it has been proved that in a great many cases of tabes mesenterica, if you retrace the history of the disease, if you go back to its first and earliest phenomena, you will find that it began with the symptoms of what has been termed remittent fever, or that the patient had enteritis or diarrhœa, which afterwards became chronic, and that then the symptoms of tabes mesenterica began to

8*

appear. In the third place, you will find that in a vast number of cases, where a fatal termination has occurred, if you pursue your dissection, and slit up the whole of the ileum, you will discover numerous old ulcerations of the mucous membrane, and find that the lymphatics which correspond with these ulcerations are in a state of manifest disease. Lastly, it has been observed, that the best treatment for tabes mesenterica, is that which is calculated to remove enteric inflammation, and that the old treatment, founded on the principle of removing obstruction by the use of alkalies, absorbents, and solvents, is erroneous and false in the majority of cases. So that we have proof of the origin of this disease in intestinal inflammation, drawn from the occurrence of analogous affections in the adult, from the phenomena of the disease in its early stage, from morbid anatomy and from treatment. I think there can be no doubt that in most instances it commences by intestinal inflammation. Of course a predisposition to disease of the glandular system will favor the occurrence. But is there no case in which the disease has commenced in the glands, and where the mucous membrane of the digestive tube is secondarily engaged? My answer to this question is, in a few cases we cannot prove that the disease commenced in the mucous membrane, and there is no reason why the glands of the mesentery should not be liable to primary tuberculous or scrofulous deposition as well as those of any other part of the body; but, in a vast number of instances, the enlargement of the mesenteric glands is secondary, and resembles the inflammation of the inguinal glands which results from chancre on the penis. I would advise you to consult the Commentaries on Pathological Propositions by Broussais. On this subject, also, Dr. Mackintosh's Practice of Physic.

There is one thing more connected with this disease, which is of considerable importance, and to which I will briefly draw your attention, and this is, that this inflammation of the glands of Peyer and Brunner, this *dothin-enteritis*, as it has been called, is a very common cause of slow convalescence in fever. You will meet with cases of fever which will go on to the 17th or 21st day, and then something like a crisis takes place; you expect that from this time forward the patient will get progressively better, but in the course of a few days you will be surprised to find no amendment, and that he is not gaining strength; you feel his pulse, and find it quick and small, his attendant informs you that he is restless at night, and when you ask him how he feels, he says he has no particular complaint, but that he is very weak, gets no sleep at night, and has no appetite. Under these circumstances you are anxious to find out what his disease is: you inquire into the state of the heart, lungs, and brain; you find no evidence of disease in any of these organs; you run over in your mind the symptoms present, the feverishness, quick pulse, want of appetite, restlessness, and finding some degree of abdominal tenderness and tympanitic swelling, you arrive at the conclusion, that the return of health and strength is impeded and delayed by the existence of a dothin-enteritis. The first person who discovered this fact was Dr. Cheyne. "In these cases," says he, "the distress of the patient often bore no proportion to the danger he was in; the former was very little, while the latter was extreme. The disease would proceed without violent symptoms; nay, a patient would seem to be recovering, although without any critical discharge; he would call for full or middle diet, and for days take his food regularly. The only circumstance in his situation which demanded attention was that he regained neither flesh nor strength, and he expressed no desire to leave his bed. Then, his pulse again became quick and his tongue dry; and he would complain of dull pain and uneasiness in his belly, attended with soreness on pressure, and a degree of fullness in the upper part of the abdomen. Then came on a loose state of the bowels and great weakness. Probably at the next visit the patient was lying on his back, with a pale, sunken countenance, and a very quick pulse; his mind without energy. Then his stools, (mucous,) passed from him in bed, and the urine also. Perhaps a hiccup came on; next his breathing became frequent, in which case death was at no great distance." In all these cases the mucous membrane and glands were found in a state of decided disease.

Now, what was the nature of this disease? It came on as a secondary affection during the course of fever, became more marked and intense, and finally destroyed the patient. I have seen very many cases of this disease. I give you this as a general rule:—when, after the apparent termination of a fever, your patient convalesces very slowly and imperfectly; when you find that he is becoming weak, that his pulse is quick, his belly tympanitic, his thirst still present, *and all this without evidence of disease in the respiratory, circulating, or nervous system*, you may suspect inflammation of the mucous glands of the digestive tube, which may terminate in deep ulcerations; and you will not be surprised if your patient should be carried off by rapid peritonitis, occasioned by an ulceration of all the coats of the intestine. I have witnessed many instances of the truth of this statement.

It has been objected to the doctrine that infantile remittent fever and tabes mesenterica depend on inflammation of the mucous membrane of the digestive tube, because it has been found that purgatives are sometimes useful in the treatment of the disease; and those who bring forward this objection ask, "if purgatives give relief, how can it be intestinal inflammation?" Now, what are the real facts of the case? These cases, which have been relieved by purgatives, are cases in which purgative medicine has been given in the early stage, and has been productive of benefit; or, in other words, where the disease is only just commencing, and where its cause is proved to be the presence of irritating matter in the bowels. A physician is called to a case of this kind; he gives a purgative; a quantity of offending matter is evacuated, and the child gets better. You should act in the very same way, and have recourse to purgatives whenever you have reason to suspect the existence of irritating or indigestible matter in the bowels. You are to employ purgatives on the same principle as every one employs emetics, in cases where corrosive poison has been swallowed; but no one is inclined to think that he will be able to cure the disease by the continued use of emetics. But unfortunately, persons do not attend to the actual state of the digestive tube; they go on prescribing purgative after purgative, until the irritation, which was originally produced only by indigestible matter, becomes exacerbated, and terminates in ulceration of the intestinal mucous surface, accompanied by all the symptoms of tabes mesenterica.—*Ibid.*—*Ibid.*

17. *Diseases of the Large Intestines*. By William Stokes, M. D.—You will see in the various systematic treatises on the practice of physic, separate descriptions of the affections of this portion of the digestive tube, you will find diarrhœa in one chapter and dysentery in another, and you will observe that a great deal of ingenuity has been expended in forming nosological differences between these affections. I fear that much of what has been written respecting them is rather calculated to puzzle and mislead than to inform the student. Viewed anatomically there is no essential difference. You may for every practical purpose place them in the same class, and consider them as the result of the same morbid condition of the same part, namely, an inflammation of the lower portion of the digestive tube. Some persons may quarrel with the term inflammation,—call it then irritation if you please, but the truth is, that it is disease of the lower portion of the intestine, the results of which are increased sensibility and altered secretion, and this description, I think, will fairly apply to one as well as the other. If a man has purging with fever and pain it is called dysentery, if he has purging without pain, and without any manifest febrile excitement, we call it diarrhœa. But in cases where persons have died, after having labored under diarrhœa for a length of time, we *generally* find, on dissection, lesions of the mucous membrane of the intestinal canal, sufficient to account for death. There are some cases indeed, in which the mucous surface takes on a gleety discharge, similar to that which follows gonorrhœa, and under such circumstances you will not be able to discover any distinct anatomical evidence of diseases. These, however, are comparatively rare, and bear little or no proportion to those cases which present distinct traces of organic lesion.—*Ibid. March* 1*st*, 1834.—*Ibid.*

18. *On Dysentery*. By William Stokes, M. D.—The first principle I have to enforce on this subject—and you may take it as an observation based on the soundest pathology—is this, that dysentery is inflammation of the large intestine. In some cases it is complicated with fever, and in others with disease in the upper portion of the digestive tube and I believe that those cases, which are termed *epidemic dysentery*, are those in which this disease is combined with typhus fever, or with an extensive affection of the small intestine—where there is ileitis as well as colitis. I shall not take up your time with discussions respecting epidemic dysenteries, or those of warm climates; it will be sufficient for the present to allude to that form of disease which is observed in this country.

I have told you that dysentery is an inflammatory affection of the great intestine, and all the symptoms during life, as well as the phenomena revealed by dissection, tend to confirm this view of the subject. We often have fever because the constitution sympathizes with the inflammation of an important organ; we have excessive pain and irritation of the intestine, in consequence of its muscular fibres being involved in the inflammation; and we have discharges of morbid, purulent and bloody secretion. You will now please to inspect this preparation and hand it round. See the effects of dysentery—the extensive inflammation, ulceration, and sloughing of the mucous membrane. Here is another preparation, you perceive the whole surface of the colon is covered with coagulable lymph, which, in some cases, forms a chief part of the dejections. Here is a preparation which exhibits extensive sloughing of the mucous membrane; its tissue, you see, is quite abraded and destroyed. Here is a preparation of chronic dysentery, which presents a very curious appearance; the mucous membrane is finely mammilated, as it were, and it is stated on the label, that the process of cicatrization was going on. If you compare it with the others. You will find a remarkable difference. Here is another specimen of dysenteric destruction.

Here, then, is a disease in which we have violent inflammation of the mucous membrane and submucous cellular tissue, and, in severe cases I believe, of all the coats of the great intestine, except the serus. Let us rehearse its symptoms briefly. Fever of an inflammatory or typhoid character, great pain and excessive irritability of the great intestine, morbid discharges of purulent, bloody, and lymphy matter, twisting pains called *tormina*, and frequently the absence of fæcal matter in the dejections.—*Ibid.*—*Ibid.*

19. *On the External Use of Croton Oil.*—This valuable drug was first made known to the profession by Dr. Connwell, in 1820, and subsequently its therapeutic effects were investigated by MM. Recamier, Bally, and Majendie; their researches were, however, limited to its internal exhibition, and it was not until 1831 and 1832 that its great value, as a counter-irritant to the skin, was clearly proved by Professor Andral.

*External Use.* With one or two fingers, or, if we choose, with a dossil of lint, wetted with the oil, we continue rubbing the skin for the space of about ten minutes. This operation should never be entrusted to the patient himself—in two cases at the Hopital de la Pitié, we observed violent ophthalmia and inflammation of the penis and scrotum induced, no doubt in consequence of the mere inadvertently carrying their fingers to their eyes and genital organs.

The eruption which is brought out by the external use of the croton oil, may be divided into five stages:—1. Rubefaction of the skin—2. Formation of vesicles—3. Conversion of the vesicles into pustules—4. Desiccation of the pustules—5. Desquamation and falling off of the crusts.

These different periods or stages are not uniformly to be observed; they are most conspicuous when the friction has been made with ten or twelve drops of the oil, on a part of the skin where there is much subjacent cellular tissue. The patient at first experiences a tingling warmth, which is quickly followed by a considerable redness, extending an inch or so beyond the sphere of the rubbing. These appearances are generally noticed within seven or eight hours, sometimes in one or two, at other times not for ten or twelve hours; the differences of time required depending, no doubt, on the delicacy of the skin. In from fourteen to twenty-six hours, myriads of small, close-set vesicles make their appearance upon the inflamed skin. Occasionally, a few of the vesicles become greatly magnified, forming true phlyctenæ, filled with a turbid lymph, which speedily change into a purulent matter. In twelve out of thirty-one cases reported by our author, the vesicles passed to desquamation without undergoing the suppurative process.

The usual period at which the serum becomes puriform, is from thirty-six to fifty-four hours after the application of the oil. In one or two days subsequently, the pus begins to exude, and forms grayish crusts over the pustules, and the desquamation is generally over by the eighth or ninth day. If the croton oil is rubbed upon any part which has been recently vesicated, the eruption is, as we might expect, more speedy and abundant.

In six cases it was tried whether the rubbing in of the croton oil, mixed with an equal quantity, or rather more, of that of almond oil, over the arch of the colon, would produce any purgative effects;—an eruption, which reached the second stage, was brought out, but the action of the bowels was not affected. Similar results were obtained when the pure oil, to the amount of twenty drops, was rubbed round the umbilicus. Dr. Rayer states that he has repeatedly induced free action of the bowels by putting two or three drops of the oil upon a surface denuded of its epidermis by a blister. We have not repeated this experiment.

*Therapeutic Effects.* The diseases in which the external use of this remedy has been employed with most advantage, are chronic rheumatism, arthritic pains, pleurodynia, paralysis, stomatitis, laryngitis, and chronic gastritis.

Case I.—*Sciatica.* A man, aged 48, was admitted into the Hopital de la Pitié on the 6th December, 1831. For five months preceding he had suffered severely from pain, beginning in his right hip, and extending down the back of the limb, along the course of the sciatic uerve to the outside of the leg. For two months and a half he was obliged to keep the house, upon then attempting to resume his work, the pain returned with all its former intensity. He attributed his complaints to exposure to wet and cold. The only treatment which had been followed before his admission was blistering the limb; but he had derived no benefit. When examined in the hospital, the pain was found to be increased by pressure, and by the heat of the bed; he complained of headache, but in other respects his general health was not amiss. Eight drops of croton oil were ordered to be rubbed in over the origin of the sciatic nerve. This produced considerable itching and redness, but no vesicles; and the pain being not relieved, eighteen drops of the oil were rubbed along the whole course of the affected nerve. Next morning the outer side of the leg was much reddened, and vesicles had formed over the trochanters. On the 11th, ten drops more were rubbed in between the trochanters. 12th. The eruption considerable—some large papulæ had appeared over the fibula. The neuralgic pain almost gone—only the heat and itchiness of the eruption are troublesome. He left the hospital in a few days quite well.

Case II.—*Sciatica.* A man, aged 50, entered the La Pitié Hospital in December, 1831, suffering from sciatica of six weeks' standing. Twelve drops of croton oil were well rubbed in between the trochanters, along the outside of the thigh, to the lower third of the leg; a copious eruption was induced, and already, upon the second day, the patient felt relieved. In six days more he was considered cured, and left the hospital, quite delighted with the rapidity of his cure.

He had experienced two severe attacks before—once in 1812, when he was treated in the Hotel-Dieu, by M. Recamier, with the essence of turpentine—at that time he was six weeks in the hospital; and again, two years ago, after exposure to wet and fatigue, he was admitted into the Hopital de la Charité, under the care of M. Fouquier, who employed blisters and friction, with anodyne balsam. He was cured then in three weeks.

Case III.—*Sciatica.* A stout, plethoric man, forty-five years of age, had for about a month felt general indisposition, frequently-returning shiverings; and neuralgic pain of the left extremity. He was taken into the La Charité Hospital, and there treated by M. Rayer with repeated venesection, the application of fifty leeches to the hip, and forty more to the back of the thigh. The essence of turpentine was administered inwardly in frequent doses; and besides all this the vapour-bath was used fourteen times. This treatment was continued for three weeks, and as little benefit had been obtained, the patient left the hospital, and a few days subsequently entered the La Pitié. At that time the pain extended from the ischium down the ankle-joint, and it was increased by walking, and by the heat of the bed: the lower part of the leg was annoyed by a feeling of formication. The digestive organs were in good order. Fifteen drops of croton oil were rubbed in over the origin of the sciatic nerve. On the following day, twenty drops more were rubbed over the tract of the affected nerve; a vesicular eruption made its appearance, and the neuralgic pain was already diminished. On the 29th, (third day,) twenty drops were again ordered. 30th. The eruption is very abundant—the vesicles have changed into large pustules. The patient complains only of the itching; the pain is gone. He remained a few days longer in the hospital, until the crusts separated; and, on the 12th day after his admission, he was discharged cured.

A case of chronic rheumatism of the shoulder-joint, supervening on typhus fever, is given, in which general and local bleeding, blisters, &c. had been fruitlessly used for the space of six weeks. The friction with a few drops of croton oil was employed twice; and on the third day the patient could move his arm—although not entirely cured, he was very much relieved when he left the hospital.

CASE IV.—*Anœsthesia, or Paralysis of Feeling.* Pierre Dumas was admitted into the Hopital de la Pitié on the 9th of Novembre, 1831. Seven months before he had an attack of erysipelas of the face, and the inflammation had extended down the left side of the neck. Three weeks after his recovery from this illness, he was suddenly seized with dimness of sight and stunning noises in his ear; these symptoms were not constant, but came and went, returning every second or third day.

This state of things lasted for about two months, during which nothing had been done in the way of medical treatment; and then there supervened a general numbness of the whole left side of the face, and the sight of the left eye became affected at the same time—the left nostril lost the sense of smell, and the left side of the mouth its sense of taste. When he was shaving, he felt as if some foreign body was placed upon his cheek; and, in chewing, the food seemed like earth in his mouth. He complained of a very severe frontal cephalalgia—the tongue was loaded, the abdomen soft, the bowels rather relaxed, pulse 90, breathing not affected. He was ordered to be largely bled from the arm—to use a mustard-bath to the feet at night, and to be put on a light emollient diet.

10*th.* No relief; half a drop of croton oil in two pills at bed-time.

11*th.* Twenty liquid stools from the pills; head-ache much better; paralysis not affected. Eeight drops of the oil to be rubbed upon the left cheek and side of the neck.

On the following day the skin was well reddened, and a large crop of confluent vesicles had made their appearance; to our great surprise he had already recovered his sight, taste, smell, and feeling. The fifth period of the eruption, or that of desquamation, was not over until the thirteenth day. He did not leave the hospital till the 12th December, having had a threatening of a relapse of the numbness in a slight degree; but this was checked by a bleeding from the foot.

CASE. V.—*Angina Laryngea—Aphonia.* An itinerant singer of the streets of Paris, presented, upon his admission, the following symptoms; a frequent, dry, and harsh cough; pain over the larynx, increased by swallowing; breathing sibilant; voice almost entirely gone, so that he could not make himself understood. Upon examining his throat, the velum and its pillars were observed red and swollen. Venesection, a sinipised foot-bath, and emollient drinks ordered. The following day he was much better; the general symtpoms were relieved; but the aphonia was as complete as before. Ten drops of croton oil to be rubbed on the front of the neck. In twenty-four hours there was a copious confluent eruption, the voice was regained, and the deglutition more easy. He left the hospital three days afterwards quite cured.

CASE VI.—*Diptherite, or Stomatitis pseudo-membranacea.* An old soldier, upwards of sixty years of age, exhibited a specimen of this disease to our notice in the Hopital de la Pitié. It had already existed for eight days, and had made considerable progress; the inside of the mouth and the surface of the tongue being covered every here and there with small oblong crusts, or laminæ of a grayish-white colour, set upon red, inflamed, and swollen bases; the submaxillary glands were painful and enlarged; the breath excessively fœtid, the lips swelled and of a purple hue; and the deglutition very difficult. Sixty leeches had been applied at two different times behind the jaws; and poultices and a multitude of gargles had been used without much good for the poor patient, who could scarcely articulate a word. Eight drops of croton oil were ordered to be rubbed in upon the sides of the neck. On the morrow a copious eruption of vesicles had appeared, and considerable relief was already experienced. From this peroid the disease appeared to have undergone a favourable change, and in fifteen days more, under the use of acid gargles and of poultices, it was altogether removed.—*Archives Gén. Aug.* 1833, *and Med. Chir. Rev. July.* 1834.—*Am. Journ. of the Med. Sciences.*

20. *On the Use of Soot in Diseases of the Eyes.*—The *Gazette Médicale*, for January, 1831, contains some facts collected by M. Carron-du-Villards favourable to the use of soot in diseases of the eyes. M. Baudelocque, physician to the Hopital des Enfans, has also extolled this article in scrofulous ophthalmia. The following is the formula of the first named practitioner:—Soot, ℥ij.; dissolve in boiling water, filter and evaporate to dryness. The residue, which is very brilliant, is to be dissolved in boiling very strong white vinegar, with the addition of 24 grs. extract of roses to ℥xij. of liquid. Some drops of this solution in a glass of water form a g od resolvent collyrium. M. Carron-du-Villards recommends granulations of the cornea to be touched with a very fine brush wet with the following mixture.—Take of Opium, ℥ij.; Cloves, ℥j.; Washed soot, ʒiv., Cinnamon water, ℥viij.; Alcohol, ℥iv. To be digested for six days in a warm place, and then expressed and filtered.—*Bulletin Général de Therapeutique, March,* 1834. —*Am. Journ. of the Med. Sciences.*

21. *Successful Treatment of Disunited Fracture by the Tourniquet.* —In a case of disunited fracture of the femur of twenty-one weeks' standing, and which resisted all the ordinary means of procuring union, has been successfully treated in St. Bartholomew's Hospital by the application of tourniquets lightly round the fractured part of the limb. Thé patient was middle-aged, and in the enjoyment of excellent health.—*Lond. Med. and Surg. Jour. Sept.* 28*th.* 1833.—*Am. Jour. of the Med. Sciences.*

22. *Luxations of the Humerus.*—M. Gerard recommends the following method for the reduction of luxations of the humerus. He states, that he has employed it successfully in every case, (eight cases,) in which he has resorted to it, of luxation of the humerus downwards, and more or less forwards or backwards. The patient being seated on a chair, an assistant placed on the uninjured side, passes his arm around the neck of the patient, and with his two hands crossed upon the dislocated shoulder, produces counter-extension. The operator placed on the injured side, raises the limb from the body, flexes the forearm on the arm, holds it, or has it held agai st the chest of the patient, an placing his left forearm under the upper portion of the luxated bone, as near as possible to the axilla, he flexes this forearm by pressing it against the patient, so that the cubital extremity of the luxated humerus is supported upon the side of the operator, who then exerts upon the luxated part a single traction, which suffices to effect reduction by replacing the head of the humerus in the glenoid cavity. It is essential to retain the inferior extremity of the luxated bone, firmly supported against the side, and as near as possible to that of the patient. In ordinary circumstances, a common man need not exert more than one-third of his strength to effect reduction, which is accomplished by a single effort, and without the patient having time to complain.—*Jour. Hebdomadaire, II.* 126, 1834.—*Am. Journ. of the Med. Scien.*

23. *Injections of Cold Water into the Umbilical Cord to Promote the Separation of the Placenta.*—Professor Hohl states that this method, first recommended by M. Mojon, will often succeed perfectly if it be used sufficiently early, and provided the vein does not contain too much blood; for sometimes cases occur in which it is not possible to dislodge the blood which the vein contains. One injection will frequently suffice. If we listen with the stethoscope over the region of the womb, where the placenta is attached, while a quantity of water is injected into the cord, a noise or rhonchus coming as if from a distance is heard; this noise is quite distinct from that of any pulsation. In favourable cases this sound becomes louder and stronger, and in addition to it, other rhonchi of a more sibilant or whistling character becomes audible. Professor H. states, that he never could hear any of these last described sounds in cases in which the placenta remained obstinately attached; they would therefore seem to be connected with the contractions of the uterus. These contractions are necessarily very imperfect, when the placenta remains fixed; and hence perhaps the absence of the sounds in these cases. Professor H. recommends that the injection of the cord be used even when it does not succeed of itself, and when therefore it is necessary to remove the placenta by manual assistance, it may be a serviceable adjunct.

The most frequent cause of failure with the injections alone, is spasmodic contraction of the uterus.—*Med. Chir. Rev.* from *Allgemeine Medic. Zeitung.*—*Am. Jour. of the Med. Sciences.*

24. *Ointment to allay the Irritation of Hæmorrhoidal Tumours.*—The following ointment is recommended by Dr. Geddings in our esteemed cotemporary, the *North American Archives of Medical and Surgical Sciences,* No. 1, as affording great relief to the irritation of hæmorrhoidal tumours:—℞. Pulv. carb. plumbi. ℥ss.; sulph. morph. gr. xv.; ung. stramon. ℥j.; ol. olivar. q. s. Ft. ung. part. applicand.

Powdered opium to the amount of a drachm may be substituted for the morphia, and if the dry white lead is not at hand, that which is ground in oil for the use of painters may be advantageously substituted. Sometimes a drachm of powdered galls may be added.—*Am. Journ. of the Med. Sciences.*

